# Capturing urban activities and environment across time scales: Object detection and time-series analysis of 6.8 million images in Accra, Ghana

**DOI:** 10.1016/j.cities.2026.107203

**Published:** 2026-10

**Authors:** Vishwa Nath, Ricky Nathvani, James E. Bennett, Sierra N. Clark, Emily Muller, Allison Hughes, James Nimo, Josephine Bedford Moses, George Owusu, Samuel Agyei-Mensah, Michael Brauer, Raphael E. Arku, Majid Ezzati

**Affiliations:** a School of Public Health, Imperial College London, London, UK; b MRC Centre for Environment and Health, School of Public Health, Imperial College London, London, UK; c School of Health & Medical Sciences, City St George's, University of London, London, UK; d Department of Physics, University of Ghana, Accra, Ghana; e Department of Environmental & Sustainable Engineering, State University of New York, Albany, USA; f Institute of Statistical, Social & Economic Research, University of Ghana, Accra, Ghana; g Department of Geography and Resource Development, University of Ghana, Accra, Ghana; h School of Population and Public Health, University of British Columbia, Vancouver, Canada; i Department of Environmental Health Sciences, School of Public Health and Health Sciences, University of Massachusetts, Amherst, USA; j Regional Institute for Population Studies, University of Ghana, Accra, Ghana; k Imperial Global Ghana Hub, Accra, Ghana

**Keywords:** Street-view imagery, Deep learning, Computer vision, Object detection, Time-series analysis, Urban analytics, Africa

## Abstract

Cities have complex dynamics on timescales from hourly and daily changes to monthly shifts and annual trends. We used time-lapsed street-view imagery (SVI) to capture and analyse temporal trends of urban environmental features in Accra, Ghana. We collected a novel dataset of 6.8 million street-view images (SVI) at five-minute intervals over five years at ten representative locations in Accra, Ghana. We used a fine-tuned YOLOv7 object detection model to detect and obtain counts of people, large vehicles, small vehicles, two-wheelers, market-related objects, refuse and animals in all images. We used a mixed-effects zero-inflated negative binomial model with indicators for hour of day, day of week, week of year, and year to consistently and coherently identify temporal patterns of object counts across time scales and sites. People and small vehicles were most prevalent in mid-morning and early evening, and market-related objects peaked in early afternoon. The number of people, vehicles and market-related objects declined on weekends at most sites, although two residential sites showed an inverse trend, peaking in all three categories on weekends. Long-term trends over the years indicate a rise in people at high-density residential sites. Over the same period, market-related objects, two-wheelers and small vehicles declined at several locations with different land-use characteristics, suggesting broad shifts in transport and commercial activity. These results demonstrate the potential of SVI and computer vision for urban monitoring to support strategies for improving mobility, traffic congestion, pollution, access to goods and services, and waste management.

## 1 Introduction

City streets are dynamic at multiple timescales. These dynamics manifest as variations in the short-term (hourly and daily) and long-term (monthly and year-on-year) movement of people and animals, different vehicles and modes of transport, and other visible objects such as market stalls. Data sources such as census data and surveys tend to focus on characteristics of residents rather than representing streetscapes, and provide limited information about these dynamics due to infrequent updates. Street-view imagery (SVI) offers a valuable resource for analysing visible features of city streets (Freitas, Berreth, Chen, & Jhala, 2023; Suel et al., 2023; Suel, Polak, Bennett, & Ezzati, 2019; Zünd & Bettencourt, 2021). Traditional sources of street view images, such as those from Google, offer good spatial coverage but are typically only updated on an annual basis. More frequently captured time-lapsed SVI can enable systematic measurement of urban dynamics in vehicle fleet size and composition, as well as the presence of pedestrians or street vending on timescales relevant for policy making (Nathvani et al., 2022; Nathvani et al., 2025). Most existing studies use images from a single point in time per location, or focus on fixed timescales such as year-on-year comparisons or hourly changes over the course of a short measurement period; multiple timescales are highly uncommon.

Africa's cities are growing faster than those in any other world region (United Nations Department of Economic and Social Affairs, Population Division, 2019) with unique patterns of development (Metzler et al., 2023; Lall, Henderson, Venables, & Avner, 2017; Metzler et al., 2025; Taubenböck et al., 2020). Our aim was to quantify the temporal dynamics of urban environmental features within a major African city on short and long timescales. We used a bespoke dataset of frequently captured SVI to study the temporal dynamics of urban environmental features at hourly, daily, monthly and year-on-year timescales in Accra, Ghana. We collected a novel dataset of 6.8 million SVIs at five-minute intervals over five years from ten representative locations. We fine-tuned an object detection algorithm using representative samples of our images and applied it to identify and classify context-relevant objects in the images, to create a temporal dataset of the number of people, different categories of vehicles, markers of marketplace activity, animals and refuse on the streets of Accra, which are key indicators related to road traffic, pedestrians and commercial activity. We then used a mixed-effects time-series model to coherently and consistently study the structure of hourly, daily and yearly changes in features of the urban environment and their long-term trends in Accra.

### 1.1 Related work

SVI captures a broad range of urban environmental features and tracers of activity involving mobility and transport, built and natural environment, commercial activity, meteorology and exposures such as air and noise pollution (Marasinghe, Yigitcanlar, Mayere, Washington, & Limb, 2024; Suel et al., 2019; Weichenthal, Hatzopoulou, & Brauer, 2019; Clark et al., 2021; Nathvani et al., 2023). Although the spatial and temporal coverage of SVI in cities vary between providers, there is consistency between them in how they capture the visual features of cities (Wang, Zhang, He, & Kada, 2025). Studies typically use street view imagery in cities, especially from sources such as Google Street View, and computer vision algorithms to extract features pertinent to a given research domain (Ibrahim et al., 2020). In particular, recent studies have used deep learning techniques.

Classification is a technique commonly used in the literature for assigning semantic labels to street view images (Ibrahim et al., 2020). Entire images are assigned specific categories or values related to the whole field of view by an algorithm, such as whether they contain sidewalks or commercial buildings (Nguyen et al., 2018), the socioeconomic status of a neighbourhood (Suel et al., 2019; Suel et al., 2023) or a street's perceived attractiveness (Dubey, Naik, Parikh, Raskar, & Hidalgo, 2016), often leveraging the high spatial coverage provided by Google Street View images. Similarly, the co-location of sensors with primary data capture of street view images has been used to classify the images taken at a given location with their corresponding ranges of air pollution concentration (Hong, Pinheiro, & Weichenthal, 2020; Nathvani et al., 2023). An analogous approach can be taken with regression to predict numerical outcomes, such as for predicting perceived safety of a street (De Nadai et al., 2016) using entire images as input.

In contrast to using an entire image, specific interpretable features such as people, vehicles and roads can also be identified, localised and quantified within individual images. If the feature of interest occurs in discrete quantities, object detection can assign each instance of an object category within an image with a bounding box (Zhao, Zheng, Xu, & Wu, 2019). Common use cases in the literature include identifying pedestrians (Yin, Cheng, Wang, & Shao, 2015), different forms of vehicles (Fan, Brown, & Smith, 2016), and street signage (Campbell et al., 2019). The presence of these objects in spatially representative images can reveal fine-grained details such as individual travel patterns (Goel et al., 2018) and local demographics in different parts of a city (Gebru et al., 2017). Image segmentation assigns each pixel within an image to a given class, separating the image into regions that occupy a certain fraction of a given field of view. This is more appropriate when the visible size of the feature is informative, such as green space (Zhang et al., 2023), road and bike lane widths (Rita et al., 2023) and sky-view in studies of urban canyons (Xia, Yabuki, & Fukuda, 2021). In addition, segmentation outputs can serve as interpretable features for use in further regression or classification, such as for air pollution (Qi & Hankey, 2021).

A significant limitation in many existing studies is their reliance on images which, though spatially representative, are captured in a narrow period of time and typically only once per location. SVI captured at regular intervals at the same location provides insights into changing environments at different timescales. For example, (Huang et al., 2024) used SVI to identify physical changes in the built environment and (Lyu, Ma, Song, Zhu, & Wang, 2023) used SVI to detect changes in green space. These studies typically operate on a timescale of years and rely on classification or regression models. Other studies have used feature extraction and shorter timescales from which interpretable changes can be measured. For instance, (Lim, Lee, An, & Park, 2024) integrated YOLOv7 for object detection with an ARIMA-LSTM model for real-time traffic volume prediction, whilst (Nathvani et al., 2022) used images captured at five-minute intervals to predict instantaneous estimates of air and noise pollution. A critical gap remains, however, in identifying complex urban environmental dynamics of cities at multiple temporal levels (hour, day, week, year). Our study aimed to achieve this by extracting objects of interest from time-lapse images captured over five years in Accra, with explicit modelling using a statistical time series model, as described below.

## 2 Methods

### 2.1 Image data collection

**Fig. 1:**
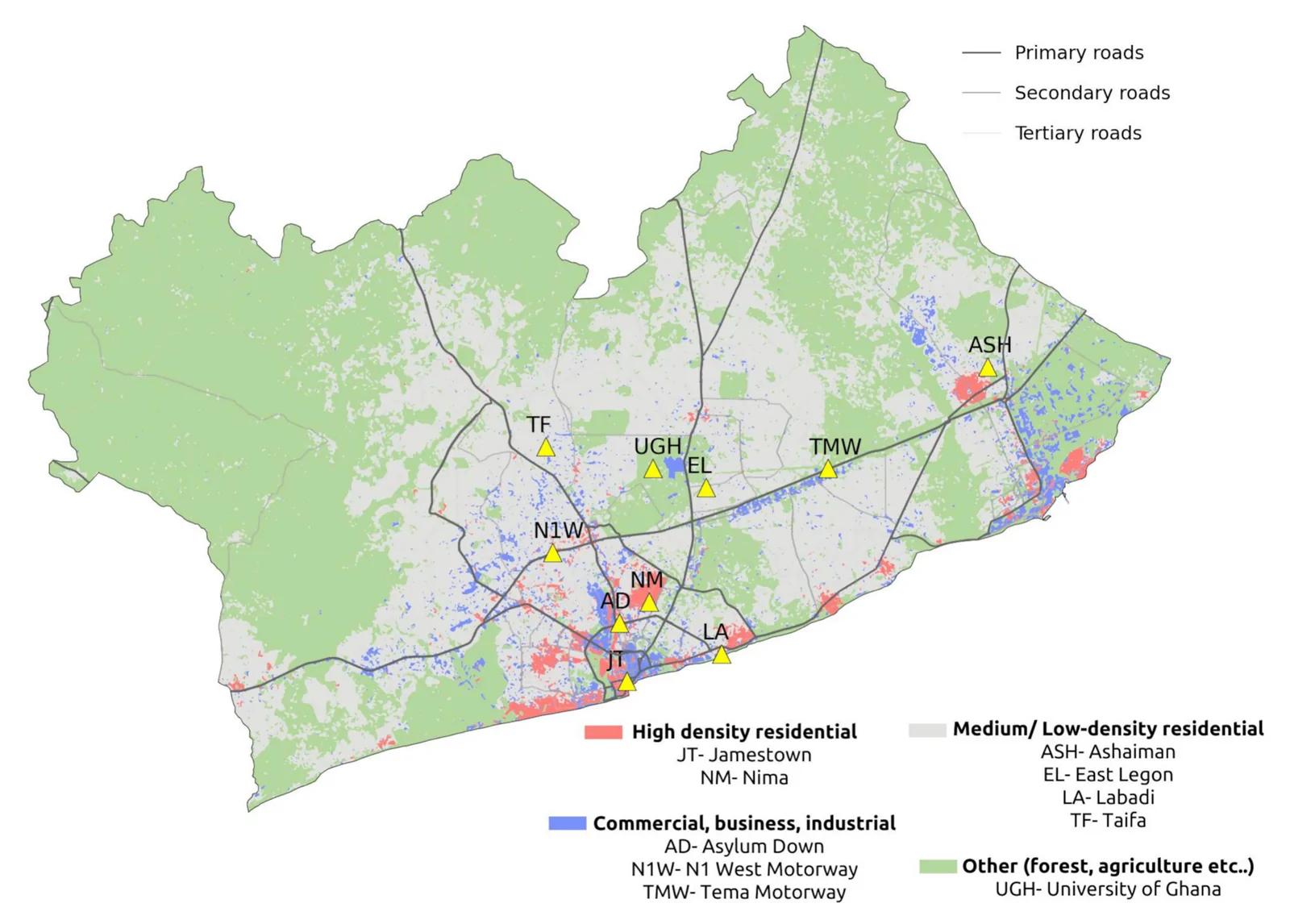
Location of ten measurement sites Ten sites marked by yellow triangles were considered for this study. (For interpretation of the references to colour in this figure legend, the reader is referred to the web version of this article.)

We collected data over a five-year period (April 2019 to March 2024) at ten fixed sites as shown in Fig. 1, across the Greater Accra Metropolitan Area (GAMA), referred to as Accra in this paper. The sites were selected to be representative of GAMA's diverse social, physical and natural environment as described in the study protocol paper (Clark et al., 2020). Areas used for site selection were classified as i) formal, mostly low- and medium-density, residential areas (four sites: Ashaiman, East Legon, Labadi and Taifa); ii) informal, mostly high-density, settlements and slums (two sites: Jamestown and Nima); iii) commercial, business and industrial (CBI) areas (three sites: Asylum Down, N1 West Motorway and Tema Motorway); and iv) other areas that are often peri-urban or rural, and can have dense vegetation (i.e., forest, grassland) or barren land (i.e., sand, soil, dirt) (Metzler et al., 2023, 2025) (one site: University of Ghana), based on classifications given in the 2014 World Bank land use map (World Bank, 2014).

**Fig. 2:**
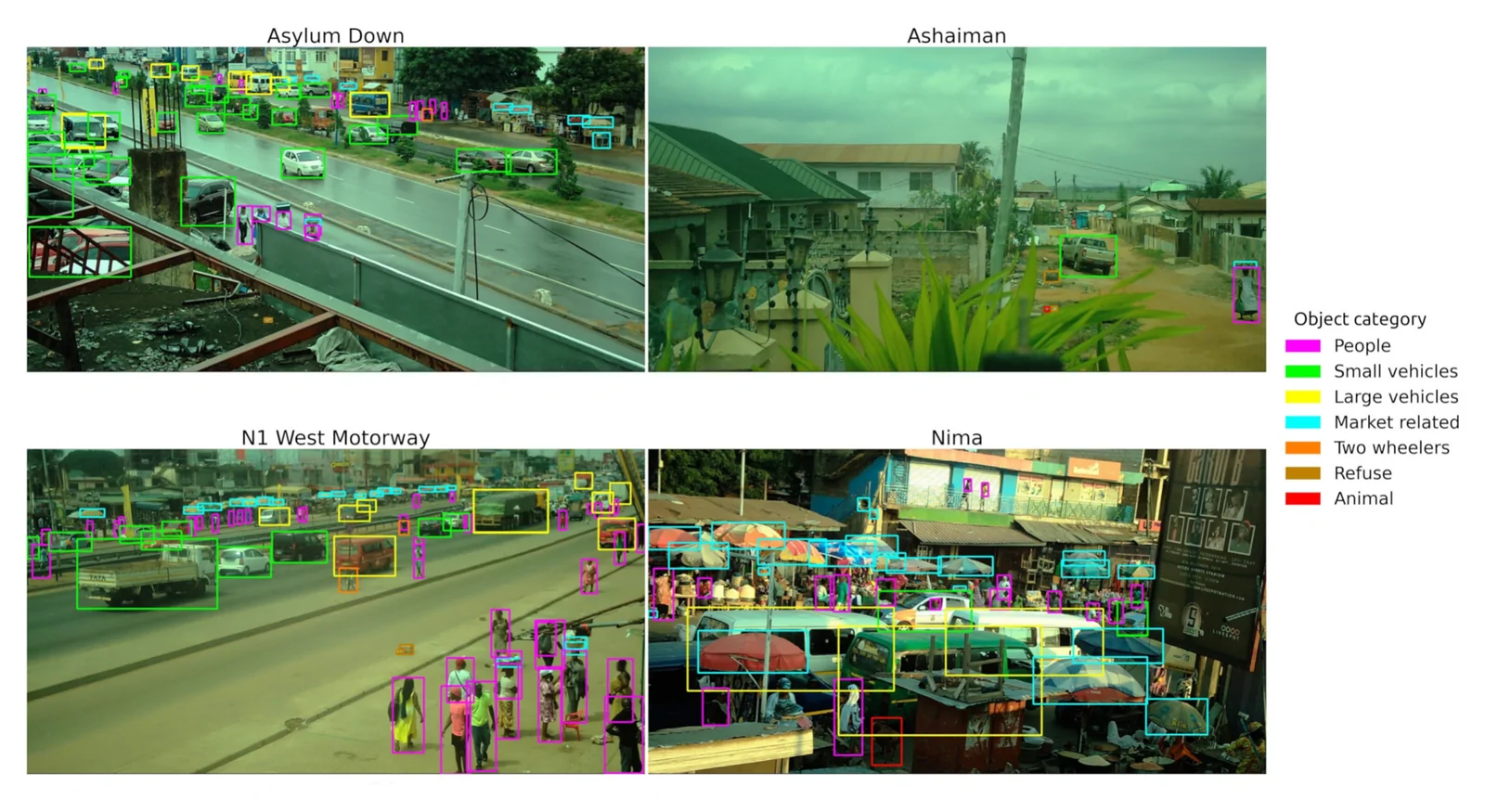
Objects identified in example images Each identified object is bounded by a box and colour-coded by a category. “Truck” refers to a pick-up truck, “bowl” refers to a cooking bowl or pot, “stall” refers to market stalls and “stove” refers to cookstoves of the type found in markets.

We deployed Moultrie-M50 cameras at each site to capture SVI. To capture the main public thoroughfare of interest, seven sites were equipped with two cameras to capture a broader horizontal view, and three sites had one camera, as seen in Fig. 2. The cameras captured images with 20-megapixel quality in a 36.7° field of view, which offered sufficient resolution for identifying features of interest from a target installation height of ~4 m. We programmed each camera to take a time-stamped image every 5 min, allowing the camera memory to store an entire week of images (~2000). We conducted weekly data downloads, followed by back-ups and uploads to the data server. Cameras automatically switched to night-vision mode when dark, and images were captured in black and white at night with infrared flash. More information on data collection, hardware and camera placement can be found in the study protocol and application papers (Clark et al., 2020; Nathvani et al., 2022).

We aimed to capture images at all target measurement sites for the entire measurement period. However, in some cases, there were equipment quality control checks, hardware failures, city lockdowns and occasional software malfunctions. The percentage of measurement campaign days during which no cameras were operational ranged from 5.6% to 39.4% (median 11.9%) across sites. Detailed information on missing images is provided in SI Fig. 2.

This environmental study was deemed exempt from full ethics review at Imperial College London and the University of Massachusetts Amherst; it was approved by the University of Ghana Ethics Committee (ECH 149/18–19) (Clark et al., 2020). The processing of the unaltered images through the application of the object detection algorithm was performed by approved researchers on access-restricted servers hosted by Imperial College London. Any images presented externally, as in Figs. 2 and 6, included the additional step of blurring human faces and vehicle license plates.

### 2.2 Objects selection and labelling in images

Twenty-four researchers from public health, data science, social and environmental sciences, and urban planning, and from countries in Africa, Asia, Europe and North America independently reviewed a subset of sampled images. They listed visual features relevant to mobility, safety, leisure and play, as well as daily activities like shopping, air and noise pollution, sanitation, and hygiene. These were further shortlisted into 20 individual types based on uniqueness, frequency and research utility: person, vendor, car, taxi, truck, bus, lorry, van, tro-tro, motorcycle, bicycle, market stall, loudspeaker, umbrella, cookstove, bowl, food, trash, debris and animal as described in detail (Nathvani et al., 2022). Tro-tros are minibuses or vans that operate on designated routes, and they are ubiquitous and the dominant form of public transport. For presentation, the 20 object types were further grouped into seven categories: people (persons and market vendors); large vehicles (lorries, vans, buses and tro-tros); small vehicles (cars, taxis and pick-up trucks); two-wheelers (bicycles and motorcycles); objects from the market and street vending (market stalls, umbrellas, cookstoves, cooking pots/bowls, food and loudspeakers) which are common in African cities (Brown, Lyons, & Dankoco, 2010; Solomon-Ayeh, King, & Decardi-Nelson, 2011), refuse (debris and trash); and animals.

The research team members manually identified and labelled these objects in bounding boxes in 1250 images. These images were simultaneously stratified by frequency and size (as measured by pixel count) of each object category in the image, and colour versus greyscale (which corresponds with day and nighttime images). These strata were split into 60–20-20% (750–250-250 image) subsets for training, validation, and testing. In this way, objects in each category were represented as evenly as possible in all three sets and under similar visibility conditions. Detailed information on object selection, identification, labelling, and image sampling can be found in an earlier paper (Nathvani et al., 2022).

### 2.3 Object detection and count extraction

Object detection algorithms predict the presence of different categories of objects in an image within multiple bounding boxes, as shown in Fig. 2. In our previous work (Nathvani et al., 2022), we used the Faster-RCNN model (Ren, He, Girshick, & Sun, 2016), a two-stage object detector that generates object region proposals followed by object classification. However, two-stage model performance is sensitive to the quality of the region proposals generated in the first stage and sometimes leads to overfitting with limited training data. Recent object detection models are capable of achieving higher recall and precision. Therefore, we performed object detection using YOLO (You-Only-Look-Once) v7 (Wang et al., 2022). YOLOv7 is a highly performant object detection model which achieves speed and accuracy in identifying objects in real-world settings relative to its network architecture size. It has a single-stage object detector architecture that divides an image into a grid and predicts the bounding boxes and object class probabilities within each grid cell. We selected a YOLOv7 model pre-trained on the MS COCO dataset (Lin et al., 2015) to use its low-level features, which have proven to be effective due to its extensive annotations and diverse object categories (Singh et al., 2024). We fine-tuned this model on our labelled dataset using transfer learning and data augmentation techniques, detailed in SI S2. Hyperparameters for the model were chosen from training on a set of 750 images and evaluating on a validation set (250 images). Subsequently, the training and validation sets were combined to fine-tune the model and were finally evaluated on an unseen testing set (250 images).

YOLOv7 offers various models, each tailored to specific requirements such as image size, inference speed, computational resources, and training time. We experimented with multiple model options, as detailed in SI S4, and the YOLOv7 E6 model was selected as most suitable for our dataset based on its performance on the test set. We also evaluated the impact of key hyperparameters, including image size and batch size, and found that two configurations of the E6 model performed well on different object categories. To leverage their strengths, we used both models, each for objects for which the model’s performance was high during testing.

The evaluation metric used for the model's performance in detecting a given category of object used in this work is Average Precision (AP), detailed in SI S2. The combination of models applied to our images achieved a mean Average Precision (mAP) of 0.594 at AP@0.5 across all 20 object categories on our test set, as detailed in SI Table 2. The highest performance was for frequently occurring object categories such as umbrella (0.78), person (0.78), taxi (0.75) and Tro-tro (0.73). In contrast, weaker performance was seen for objects such as vans (0.054), debris (0.083) and street vendors (0.13). Cookstoves (0) were not detected, likely due to extremely low counts. The performance of the final consolidated model on day (colour) images was slightly higher than that of nighttime (greyscale) images, with mAP of 0.60 as compared with 0.58, weighted for object frequency, as shown in SI Tables 3 and 4. Objects like taxi (0.84) and loudspeaker (0.83) performed well in daylight conditions, even in the case of the latter which was sparse in our training data. While large objects like bus (0.995) and lorry (0.79) were easily detected, others like street vendors, bicycles, cookstoves and loudspeakers dropped to zero at night, either because they were less frequent at night or because they became less visible in low light conditions. Refuse detection improved in nighttime images (0.39) compared to day images (0.17) which may result from trash being less obscured by other objects, such as people, at night. A significant positive correlation existed between the average visual size of an object in the images and its detection accuracy, as detailed in SI Table 5, with performance increasing from 0.11 for small objects, 0.39 for medium objects to 0.51 for large objects. Further details of the model’s performance in detecting individual object types is detailed in SI Figs. 3 and 4, in the form of confusion matrices and Precision Recall (PR) curves.

The final model was applied to all images obtained from our data collection campaign, yielding the counts of objects in each image. To reduce sensitivity to high variance fluctuations in object counts in five-minute intervals, we calculated the sum of all counts recorded within each hour for each site and day. Where two cameras operated at a given hour, the hourly counts across the two cameras were averaged so that the site was not over-represented. After adding counts within each hour, we obtained 356,383 hourly counts of each object category across 6.8 million images taken at ten sites.

### 2.4 Statistical modelling of object counts over time

We quantified the associations of the object counts at each site with variables representing timescales of hours, days, weeks and years over the study period. We included different timescales in our model to investigate the short and long-term dynamics of objects, e.g., daily commuting patterns as well as year-on-year changes in traffic volume and fleet composition.

We used mixed-effects models to estimate the hourly counts of each object at each site and also to account for how object counts vary over time. Object counts were modelled as a function of hour (0–23), day (of week, 1–7), week (of year, 1–52) and year (2019–2024), using a mixed-effect, zero-inflated negative binomial regression. The zero-inflated negative binomial model is appropriate for our count data, which have a variance greater than the mean count and which contain excess zero counts (Cameron & Trivedi, 1998). We conducted all analyses separately by site and object to differentiate trends that may differ between objects and sites, and for computational feasibility due, in part, to the large number of object counts.

Specifically, the hourly counts of an object, *y*, at time *t* were modelled separately for each individual object category at each site as: y_t_ ~ Zero-inflated Negative Binomial (μ_t_, ϕ) with the zero-inflation probability: π = logit^−1^(γ_0_)

and

log(μ_t_) = β_0_ + β_1_·year_t_ + β_2_·week_t_ + β_3_·day_t_ + β_4_·hour_t_ + β_5_· (hour_t_ × day_t_)+ u_p_

where μ_t_ is the mean count at time *t, ϕ* is the dispersion parameter, π is the probability of zero-inflation, γ_0_ is the estimated inverse logit of π, β_0_ is the overall intercept, β_1–5_ are fixed effects quantifying temporal deviations from the overall mean, and u_p_ is a normally distributed random intercept term for each observation period *p*.

The logarithmic specification characterises variation over time in relative (versus absolute) scale. β_0_ quantifies the overall intercept across time. β_1_ measures the deviation from β_0_ due to change in a year, whilst β_2,_ β_3_ and β_4_ do the same for week of year, day of week and hour of day, respectively. β_5_ is an interaction term between hour and day, which allows the hourly trend to be different for each day of the week. To present trends separately across hours of the day and days of the week (see Figs. 4 and 5), we averaged the multiplicative effect of the interaction term β_5_ over days of the week and hours of the day, respectively. The u_p_ are random effects, with distribution u_p_ ~ (0, σ^2^) where *p* indexes each download period of the data (typically weekly). Their inclusion accounts for variations in object counts between download periods due to small changes in camera angle and the number of operational cameras (one or two) across sites. Additionally, the u_p_ account for one-off events such as processions, building fires, public and religious holidays or celebrations, which can disrupt or enhance mobility, potentially leading to variations in the observed counts.

We implemented models using the glmmTMB library, separately for each site and object category. Each model was fit using the default optimiser in glmmTMB to minimise negative log-likelihood until they converged or for a maximum of 100 iterations. We evaluated model performance by calculating the average R^2^ achieved across five-fold cross-validation. Average R^2^ exceeded 0.75 for most object and site combinations. Models trained to estimate counts of animals, large vehicles and refuse performed worse than those for other objects, as shown in SI Fig. 6, which we attribute to their generally low counts as recorded from object detection in our images, as shown in Fig. 2. To evaluate whether the inclusion of β_5_, the interaction term between hour and day, improved model fit, we conducted five-fold cross-validation, training and evaluating models with and without the interaction term for identical folds. As seen in SI Fig. 6, most points lie above or close to the diagonal line, indicating that the inclusion of the term led to an improvement in fit quality and therefore we decided to include such a term in our final results.

To determine the trend in object counts across years, we calculated the Spearman correlation of the year effects (*β*_*1,2019*_, *β*_*1,2020*_, etc.) with year. This measures the strength and direction of the monotonic relationship between the year and the estimated object counts. To estimate the uncertainty associated with each Spearman correlation, we used bootstrapping. Bootstrap samples for each of the elements of β_1_, the effects of the years 2019–2024 on an object type at a given site, were drawn from the distribution given by their estimated means and standard errors. We created 10,000 sets of samples for each object type at each site. For each set of sampled β_1_, the Spearman correlation coefficient is calculated, resulting in 10,000 bootstrapped coefficients. The 2.5th and 97.5th percentiles of these 10,000 coefficients give the uncertainty interval for that object and site combination. These are shown in Fig. 7 of our results with trend lines.

## 3 Results

### 3.1 Overall counts of objects

**Fig. 3:**
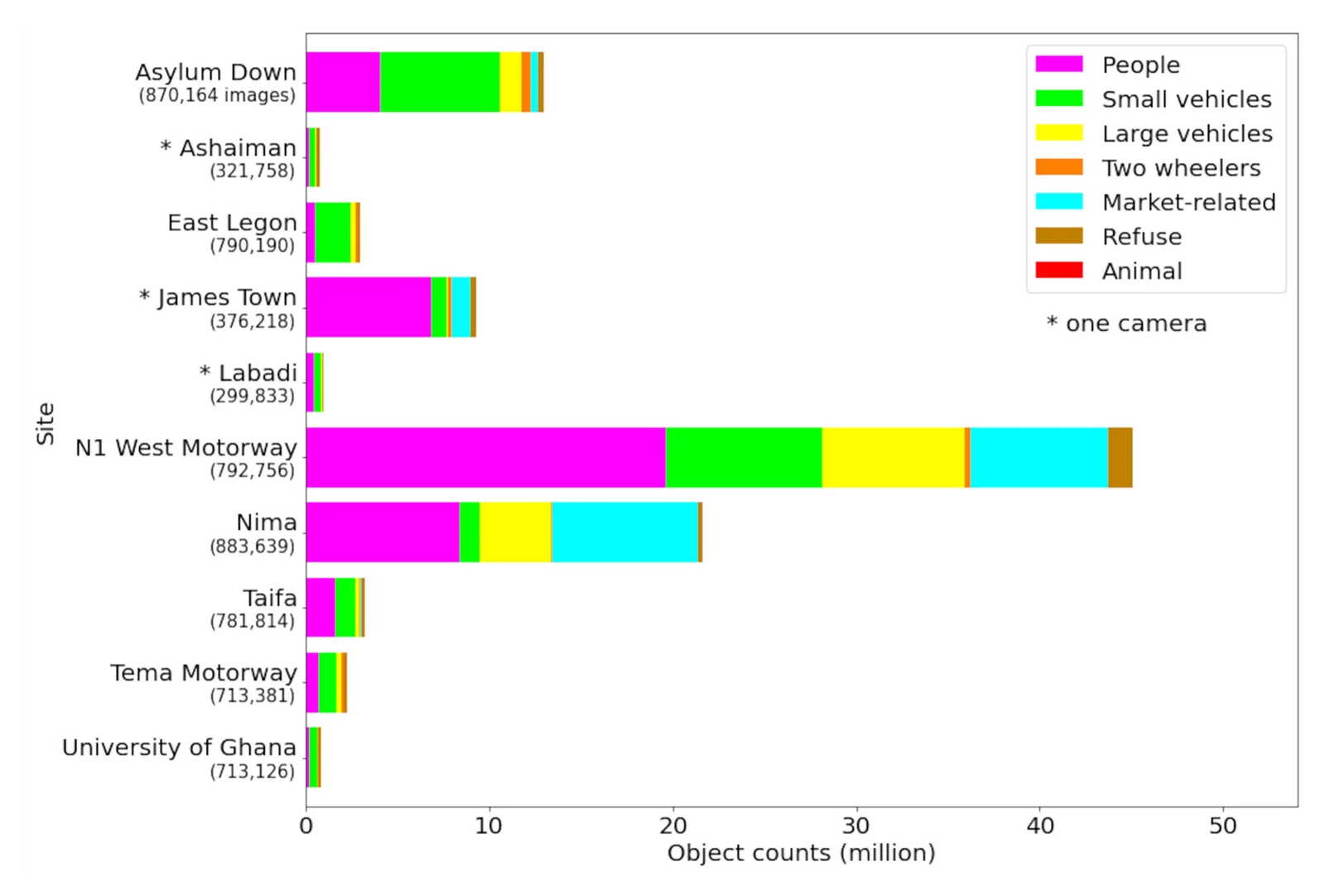
Object counts by site and object category The stacked bar plot shows the total number of objects recorded from each category, extracted from the entire dataset across all sites. The numbers after each site's name show the number of images from that site. Three sites marked by * had one camera, while the other sites had two cameras.

Fig. 3 shows the counts of all recorded objects at each site. We identified more than 100 million occurrences of the 20 target objects in our 6.8 million images. Of these, 42.05 million (41%) were persons, followed by cars (17.67 million; 18%), umbrellas (14.97 million; 15%) and tro-tros (12.05 million; 12%). The least common objects were stoves (20,799; 0.02%), animals (46,038; 0.05%), loudspeakers (61,100; 0.06%) and stalls (95,963; 0.09%).

The number of objects recorded during the measurement period varied from site to site, primarily based on the land use and activity level at these locations. For instance, the three sites with the most overall objects, N1 West Motorway (45.09% of all objects), Nima (21.65%) and Asylum Down (12.97%), exhibited a high number of people, vehicle traffic and daytime marketplace activity (Fig. 3). These sites all had two cameras, which captured a larger effective field of view, though the site with the next highest number of objects, James Town (9.31%), contained only one camera. The overall object counts in N1 West Motorway (45.09%) were significantly higher than in Tema Motorway (2.28%), even though both are motorway sites with two cameras. This may be because the N1 West Motorway is located near a traffic signal in a densely populated residential area and has roadside shops and vehicle stoppages, attracting more road vendors and higher activity than the Tema Motorway, which is situated on the city's outskirts with few buildings or amenities to attract people or vehicle stopping (Nathvani et al., 2025). People and small vehicles were the most populous object categories at all sites, except Nima and James Town, both informal and densely populated settlements, where market-related objects were more common than small vehicles. At both N1 West Motorway and Nima sites, the higher proportion of large to small vehicles compared to other sites may result from their locations as routes of tro-tro transit.

### 3.2 Association of hour of day with object counts

**Fig. 4:**
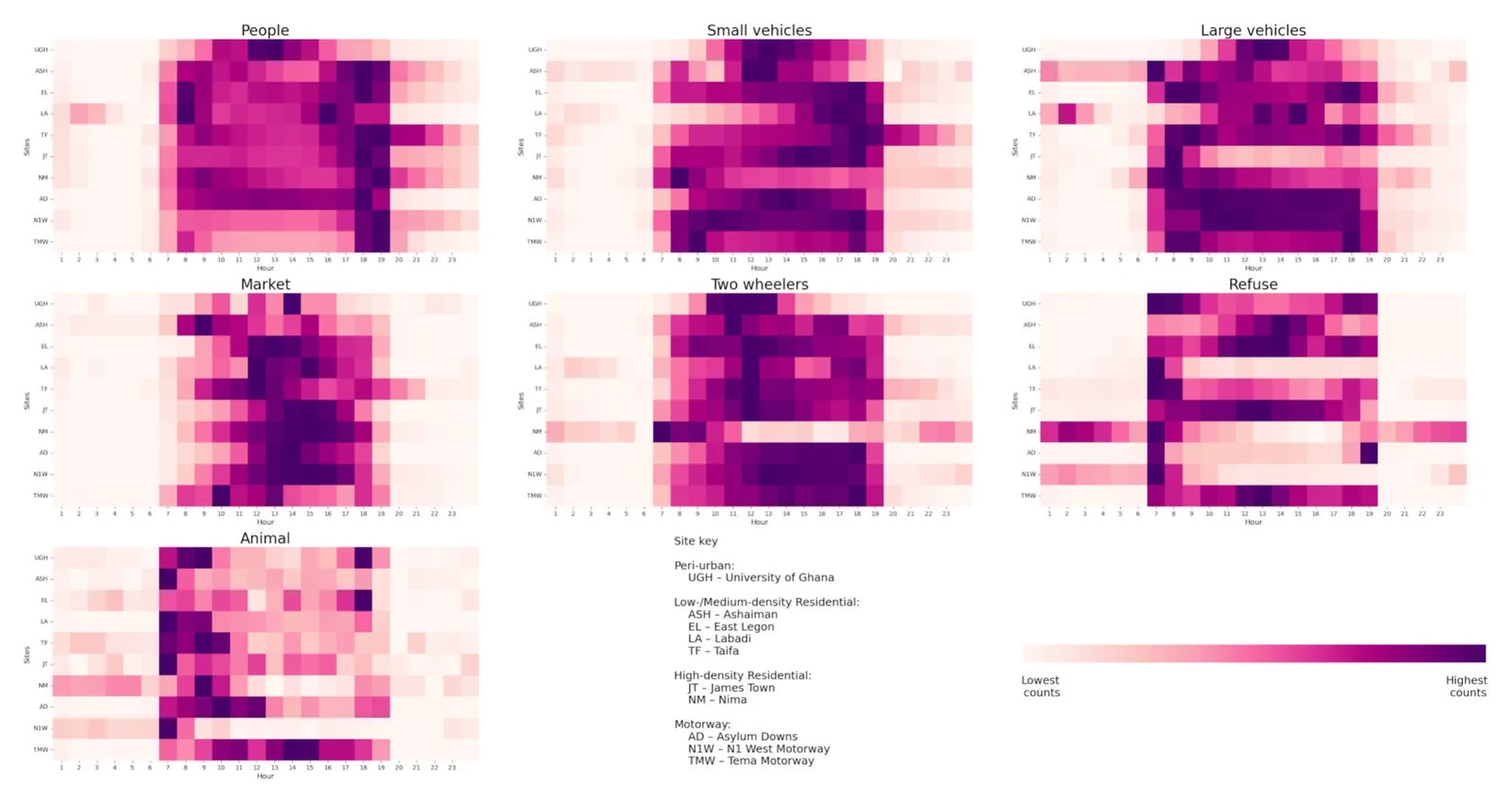
Association of hour of day with object counts The heatmap shows the coefficients for different hours of the day for combinations of object categories and sites from time series models described in Methods. Each row in each panel shows how the object count at that site varied by hour of the day. Due to differing counts across objects and sites, we rescaled the coefficients to enable comparison across hours of the day for each site and object, independent of other sites or objects. To allow comparisons across objects and sites, for each combination of object and site, the coefficients were scaled to range from 0 to 1 by subtracting the minimum count across the 24-h coefficients and dividing by the difference between maximum and minimum counts.

Fig. 4 illustrates the association of object counts with times of day (hour). The presence of people at most sites was bimodal, peaking in the mid-morning and early evening hours, which coincided with commuting times and activities such as church services in the morning or shopping and roadside food stalls in the evening. The counts of people following the mid-morning peak declined gradually, whereas the early-evening peak saw a rapid drop-off into the night at most sites.

Small vehicles, including cars, taxis and pick-up trucks also tended to peak both in the morning and evening for half of the sites, with the remaining sites exhibiting a more unimodal peak in the afternoon; there was no clear distinction in site type to distinguish the two behaviours. Sample images from Asylum Down in Fig. 6c showed a greater presence of small vehicles during noon and evening compared to other times. However, in Ashaiman, James Town and Asylum Down, which fell under different land-use types, the number of small vehicles peaked in the early afternoon, preceding the evening peak in the presence of people at those same sites.

The hourly patterns for large vehicles, including lorries, vans, buses and tro-tros, were irregular between sites with some exhibiting morning peaks, as in Ashaiman, James Town and Nima, whilst others more closely followed the respective site-specific trends for small vehicles. These variations may be due to factors like delivery schedules, shipment patterns and established pickup routes and should be explored in further in-depth quantitative and qualitative studies. Large vehicles were present consistently from dawn to dusk at the N1 West and Tema Motorways. Unlike small and large vehicles, at six of the nine sites, the number of two-wheelers peaked in the middle of the day, from ~11:00 to ~13:00. This differs from the peak times for small and large vehicles at those same sites, particularly in most of the residential sites, and may be due to motorcycles used for online food delivery (Pochet & Lesteven, 2025) or ride hailing (Abdul-Karim, Adumpo, & Akaateba, 2026; Oteng-Ababio & Agyemang, 2012). Other reasons may include a preference for travel times that are not dominated by larger vehicles on the road in the interests of safety, though some two-wheelers as detected in our images might also be obscured during times of peak traffic.

At all sites, market-related objects were broadly unimodal in presence, tending to peak in the early afternoon, except for Ashaiman and Tema Motorway, where their presence was highest in mid-morning. This pattern may be partly because vendors often commute from distant locations using inconvenient transport and need to return home before nightfall with the goods that they sell (Anderson et al., 2024; Dumedah, 2017). Refuse, which includes trash, showed less distinct hourly patterns, possibly because refuse waste generation rates and cleaning schedules both influence accumulation and because visibility may be affected by the presence of other objects. Animals were detected during the daytime at most sites due to pets like dogs and cats roaming freely, as well as domestic livestock, particularly free-range chickens (Aboe et al., 2006), scavenging for food when not deterred by human and vehicle presence.

The heatmap also reveals that the University of Ghana experienced distinctly reduced activity levels at night compared to day, with the presence of all objects except animals and refuse peaking in the middle of the day. This pattern is likely due to the site's proximity to an administrative building, resulting in high activity during office hours.

### 3.3 Patterns by day of week

**Fig. 5:**
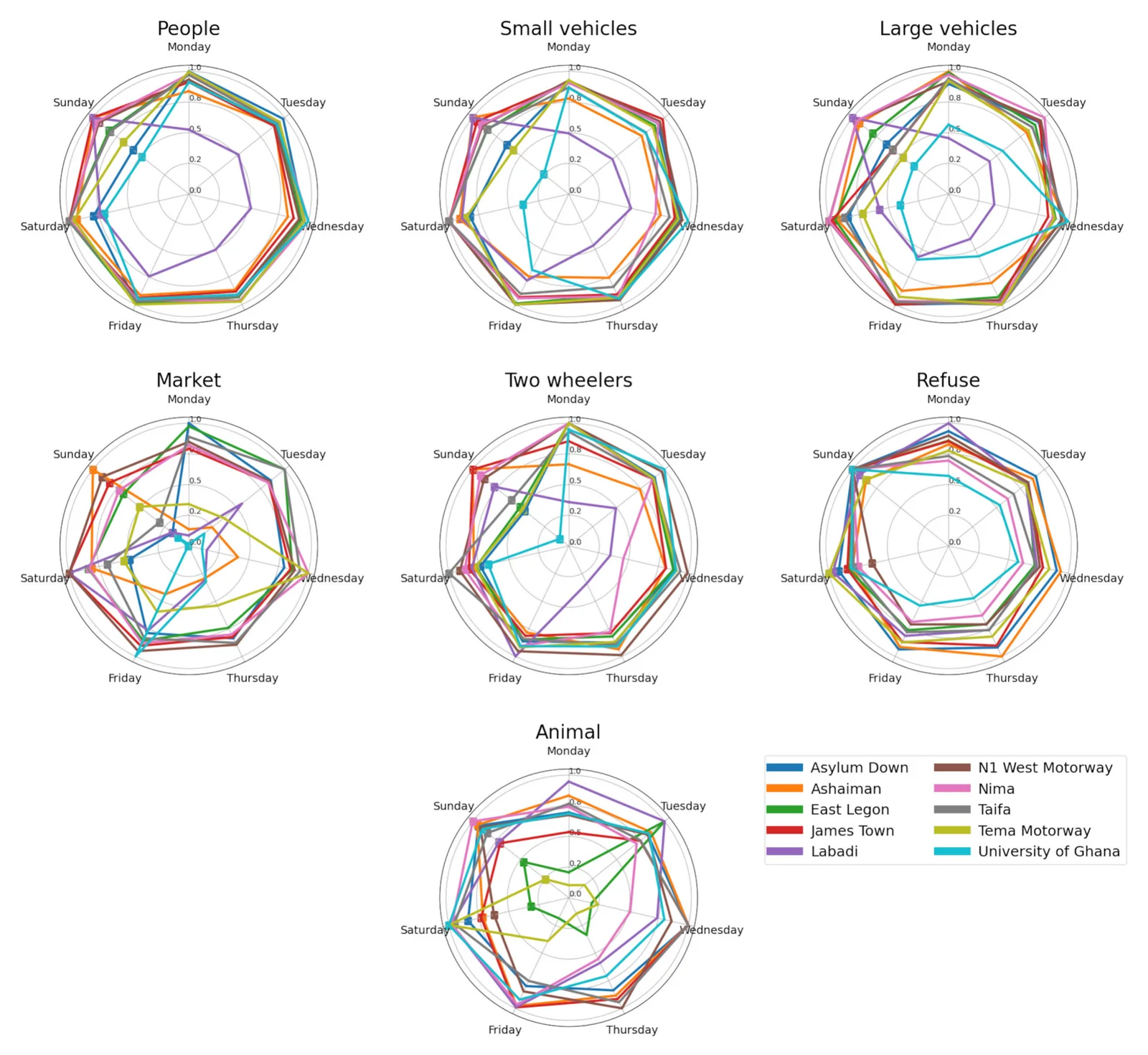
Association of day of week with object counts The radar plot shows the coefficients for different days of the week for distinct combinations of object categories and sites from the time series models described in Methods. Each line in each panel shows how the object count at that site varies by day of the week. To allow comparisons across objects and sites, the coefficients were scaled to range from 0 to 1 for each combination of object and site, by subtracting the minimum count across the seven day-of-week coefficients and dividing by the difference between maximum and minimum counts.

**Fig. 6:**
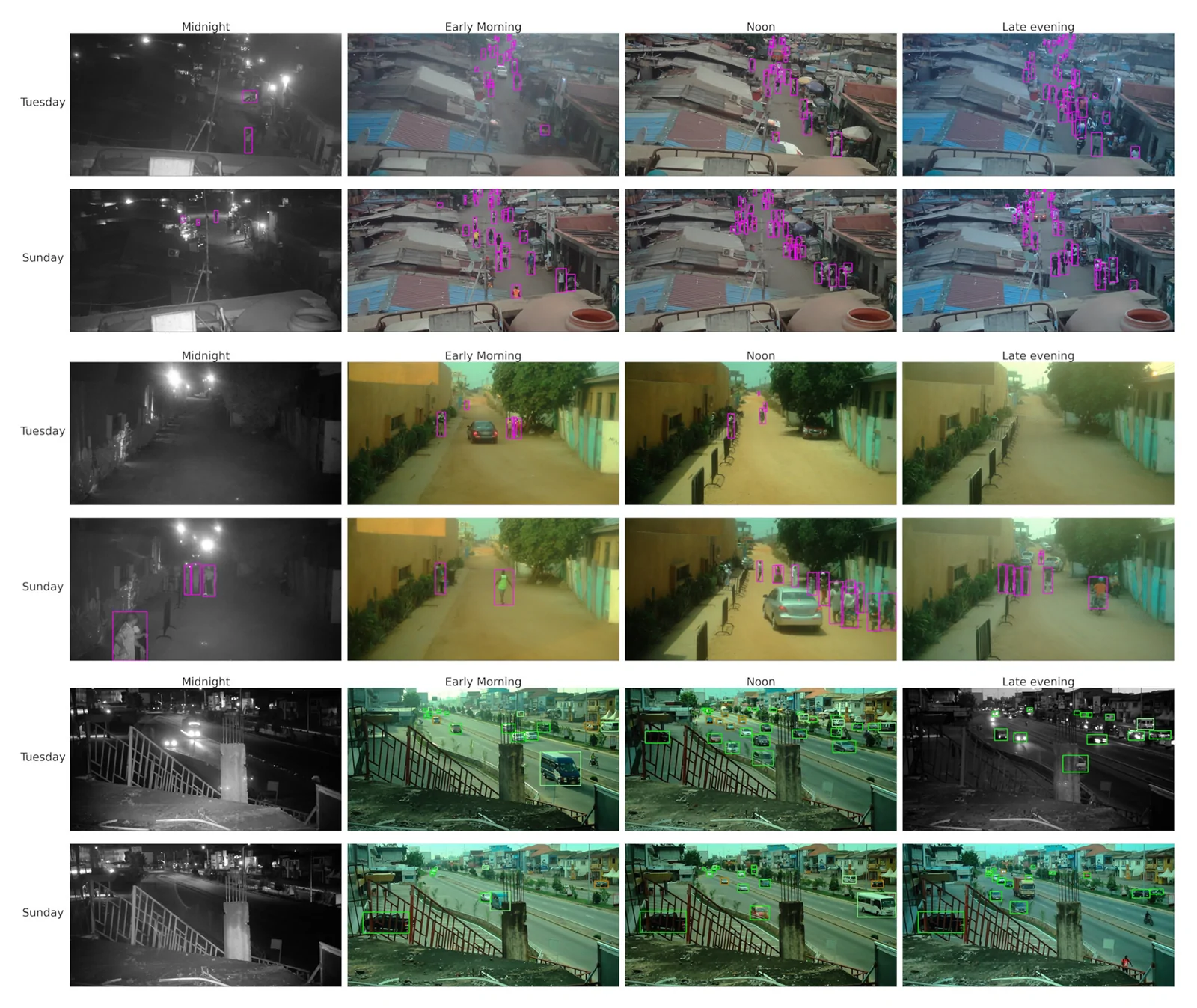
Examples of object variations detected at different times of day and day of week on a randomly selected week. A. People category at James Town. B. People at Labadi. and C. Small vehicles at Asylum Down.

Fig. 5 shows the variations in object counts across days of the week. Most sites exhibited similar patterns for people and all vehicle categories, with high activity levels on weekdays and relatively low levels on weekends. For example, sample images from James Town (Fig. 6a) show a greater presence of people on Tuesday compared to Sunday. An exception to the weekday-weekend pattern was Labadi, whose pattern was reversed and had the highest counts of people, vehicles and refuse on Sundays. This pattern can be further visualised in the sample images in Fig. 6b, which show greater object counts, particularly people, on a Sunday compared to Tuesday. This is likely a result of its proximity to a church, a roadside food stall and a public leisure beach. The relative difference between sites with respect to site-specific highest daily counts was on Sundays, commonly the busiest or sparsest in a given object category.

Market-related objects, primarily umbrellas, exhibit distinct peaks on specific days at some sites including at Ashaiman (Sunday), Tema Motorway (Wednesday), Labadi (Saturday) and University of Ghana (Friday). This may be because people use umbrellas for shade and also street vendors come to sell their food, often with stalls shaded by umbrellas, on days that specific activities like church services and markets take place. The presence of refuse saw comparatively slight variation over the week across all sites relative to other object categories.

### 3.4 Trends of object counts over the years

**Fig. 7:**
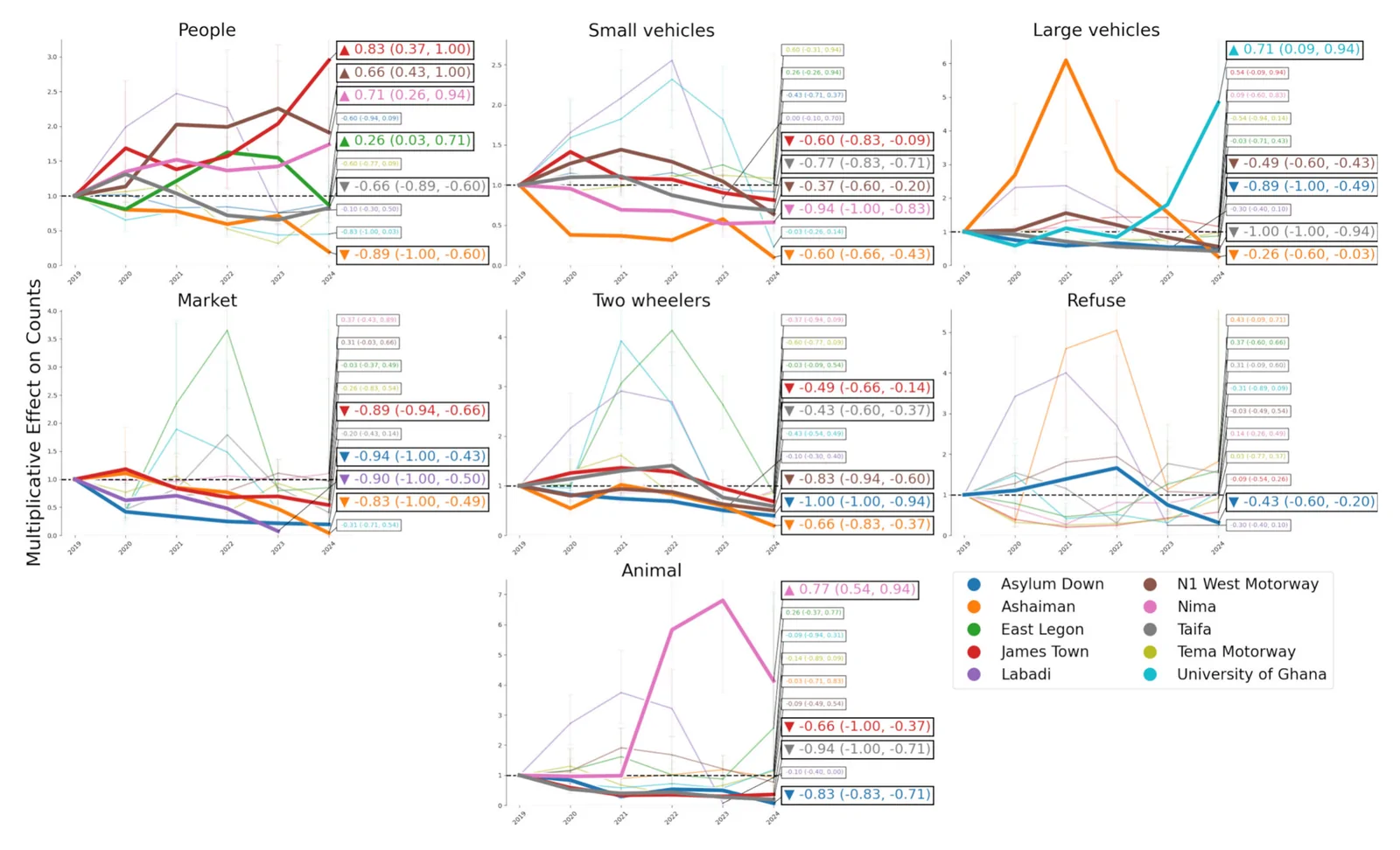
Association of year with object counts The line graph shows the coefficients for different years for distinct combinations of object categories and sites from time series models described in Methods. Sites whose 95% confidence intervals for a Spearman rank correlation between year and coefficient values exclude zero are highlighted in bold font, and the trend direction is indicated with an arrow.

Fig. 7 shows how object counts changed on average from year to year, relative to 2019. Both high-density residential sites, James Town and Nima, had rising trends in people over time, along with the motorway site of N1 West. Conversely, two low- and medium-density residential sites, Ashaiman and Taifa, showed a decreasing presence of people on streets over time. Market-related objects declined at most sites, which may indicate a shift towards more formal shopping options like supermarkets (Reardon, Timmer, Barrett, & Berdegué, 2003; Yawson & Yamoah, 2022). This trend may possibly be due to the growing use of online shopping since the COVID-19 pandemic (Asante & Mills, 2020; Farrell, Wheat, Ward, & Relihan, 2020), as observed in other countries and consistent with Ghanaians' increasingly positive consumer sentiment towards e-commerce (Jibril, Kwarteng, Pilik, Botha, & Osakwe, 2020; Turkson, Amoah, & Amoah, 2026). All vehicles showed a notable negative trend at most of the sites. This may be due to the development of new connecting roads in GAMA, aimed at reducing congestion inside the city (Amedzro, Essien, Issah, & Owusu, 2024). Sites like Asylum Down, Ashaiman, James Town and Labadi all showed a decline in the presence of these objects over the years. Three sites showed a slight decrease in animals across the years, which may have resulted from restrictions by local government and educational campaigns against animal loitering. However, Nima was an exception, which saw a significant rise in animals seen in images after 2021; the reason for this trend is not known.

### 3.5 Trends of vehicle fleet composition over hours of day and years

Transport in Accra is characterised by high dependence on motorised transport (Agyemang, 2017). The distribution of the vehicle fleet on main thoroughfares is crucial for understanding and improving mobility patterns and reducing congestion and environmental impacts such as air and noise pollution. Fig. 8 shows the average share of different vehicles observed in each year. In general, the share of cars either steadily increased or remained relatively constant across sites. In contrast, the proportion of tro-tros decreased at motorway sites. This combination of trends may indicate a shift in usage from public transportation towards private cars. Additionally, the proportion of vehicles that resemble traditional taxis declined over the years. The use of private cars as app-based taxi services has significantly risen (Abdul-Karim et al., 2026; Boateng, Appau, & Baako, 2023), contributing to the overall increasing trend of cars relative to taxis, as these app-based taxis are indistinguishable from privately owned cars in our images. The diurnal pattern of vehicles in Fig. 8 reveals that taxis were primarily operated during daylight hours (6 AM - 6 PM). In contrast, tro-tros were prevalent throughout the day, which are widely used as public transport from pre-dawn to late-night. The presence of tro-tros during the night is likely due to parking on the streets, as seen in a manual audit of a sample of our images.

## 4 Discussion

By leveraging a novel dataset of 6.8 million SVI captured at five-minute intervals over five years in combination with object detection and time-series modelling, we investigated interpretable temporal patterns of seven categories of key objects related to urban livability, safety, health, and sustainability. Our findings revealed differences in pedestrian, vehicle and marketplace activity at multiple timescales. This underscores the potential of SVI and computer vision techniques integrated with time-series modelling as a powerful tool for urban analysis, particularly where cities are changing at faster rates than administrative data is capable of measuring. Additionally, our previous work has shown that levels of air and noise pollution can be linked to the presence of different vehicles and tracers of market-place activity over time (Nathvani et al., 2022). This information can enable city officials to monitor change, identify emerging trends, and develop more effective strategies to address challenges such as mobility, pollution, traffic congestion, access to goods and services and waste management, ultimately creating more sustainable, equitable, and inclusive cities.

Our results provide empirical evidence or indications of several key trends. Sites exhibited a dichotomy of growing and diminishing footfall over the years, which may require a differentiation in policies aimed at supporting growth in certain areas through the use of infrastructure and congestion management (Amanor, Adanu, Adams, & Adi, 2024; Imoro Musah, Peng, & Xu, 2020) whilst reconsidering land use and supporting places whose activity is declining. Reduction in the visible presence of informal marketplace activity may indicate shifts in patterns of commerce and economic activity of Accra (Beek & Thiel, 2017; Brown et al., 2010) and other African cities (Ligthelm, 2006; Onyebueke & Geyer, 2012).

Differences in the temporal patterns of vehicular activity, such as changes in the number of different vehicles which indicate potential shifts towards private car ownership or the use of ride-hailing apps, with declining proportions of tro-tros and traditional taxis, offer insights for transport policy (Birago, Opoku Mensah, & Sharma, 2017; Imoro Musah et al., 2020), such as investments in more efficient public transport options (Agyemang, 2015; Anderson et al., 2024; Ministry of Transport, 2016). The presence of two-wheeled vehicles, which include bicycles and motorcycles, at times out of sync with other vehicles, highlights the need for better safety measures (Tuffour & Appiagyei, 2014), such as dedicated bike lanes especially as their use for personal travel and ride-hailing increases (Abdul-Karim et al., 2026; Oteng-Ababio & Agyemang, 2012). Furthermore, the fleet composition on roads and its change over time is a critical factor in determining how lane usage and the addition of new roads need to be adapted as the presence of two-wheelers, buses and private cars changes on roads. Choices, such as whether implementing bus rapid transit systems might alleviate urban congestion, depend on the data of road usage by different vehicles at peak hours, which our modelling provides (Bertaud, 2018).

The hourly patterns indicate a persistent presence of waste at some locations. Inefficient waste management can lead to a considerable increase in waste volumes in public areas. This results in the accumulation of insects and pest manifestations. Poor solid waste management has significantly contributed to flooding in Accra, as waste disposal clogs major storm drains and gutters, creating environmental hazards (Fagariba & Song, 2016).

### 4.1 Limitations

Due to limited resources for maintaining the continuous operation of the cameras, we were limited to ten locations in Accra, inhibiting a comprehensive spatial analysis as in previous work which collected data over one year only (Nathvani et al., 2022). Nonetheless these sites were representative of Accra's diverse social, physical and natural environment (Clark et al., 2020). The completeness of object count at any given time is limited by coverage of the camera's field of view (Fan et al., 2025) and can be obscured by parked vehicles or vegetation. We accounted for systematic differences in camera features such as angle and the number of operational cameras, through the use of a random intercept term for each camera's download period. The performance of the object detection model may be impacted by factors like low-light conditions, particularly during the night when images are captured in greyscale. To overcome this, we employed a variety of data augmentation techniques during training the object detection models. This study did not explicitly account for weather conditions (e.g., rain or dust storms), processions, national holidays, and unusual events, which could influence the object counts. However, by including random intercepts indexed by download period in the time-series model, we limited the influence of these events while still allowing the underlying time pattern to be captured by the fixed effects. Additionally, the performance of the object detection model can be variable, potentially resulting in missed detections of certain objects in the images as detailed in SI Fig. 5. Finally, stationary objects may be counted multiple times within the same hour, leading to a greater perceived prevalence in the data, despite corresponding to a single instance of the object.

## 5 Conclusion

Our analysis shows that SVI at fine temporal resolution over the period of many years is capable of measuring and monitoring changes in the rapidly evolving African cities. Our analysis in Accra revealed changes in pedestrian, vehicular, marketplace and even animal behaviours on the timescales of hours to years, indicating the complex and spatially multifaceted nature of Accra's dynamics and growth. Given the scalability of this approach, we see great potential for the use of time-lapsed SVI, which may be obtainable from the growing use of CCTV (Jacobs, 2021; Jili, 2022) in SSA cities in supporting data-driven urban policymaking and governance, provided appropriate safeguards for privacy as used. Developments in edge computing methods and advances in object detection algorithms may allow such metrics to be calculated on site without the need for storing potentially sensitive imagery (Huang et al., 2022; Ren et al., 2016).

## Supplementary Material

**Appendix A.Supplementary data**

Supplementary data to this article can be found online at https://doi.org/10.1016/j.cities.2026.107203.

Supplementery Information

## Figures and Tables

**a:**
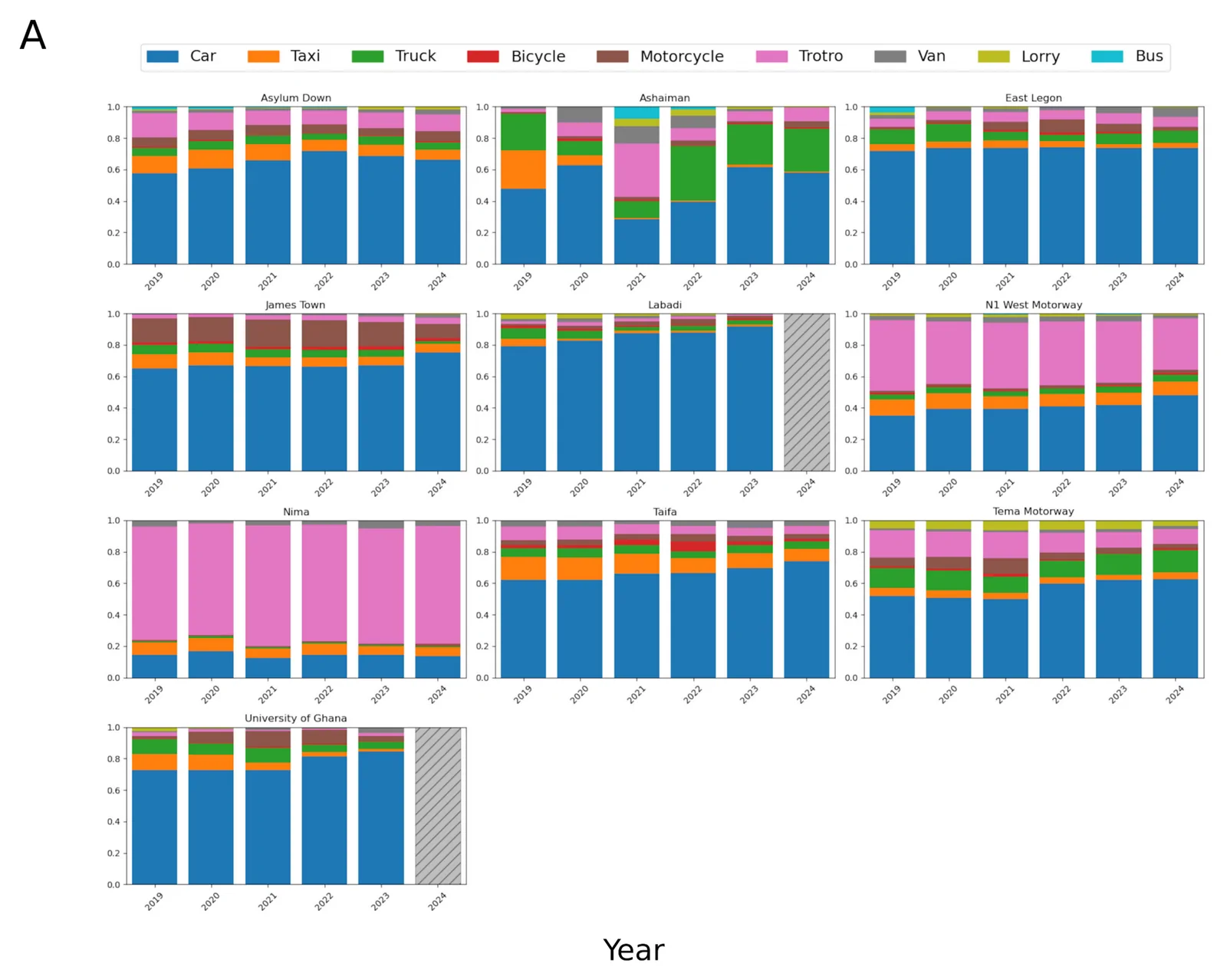


**b:**
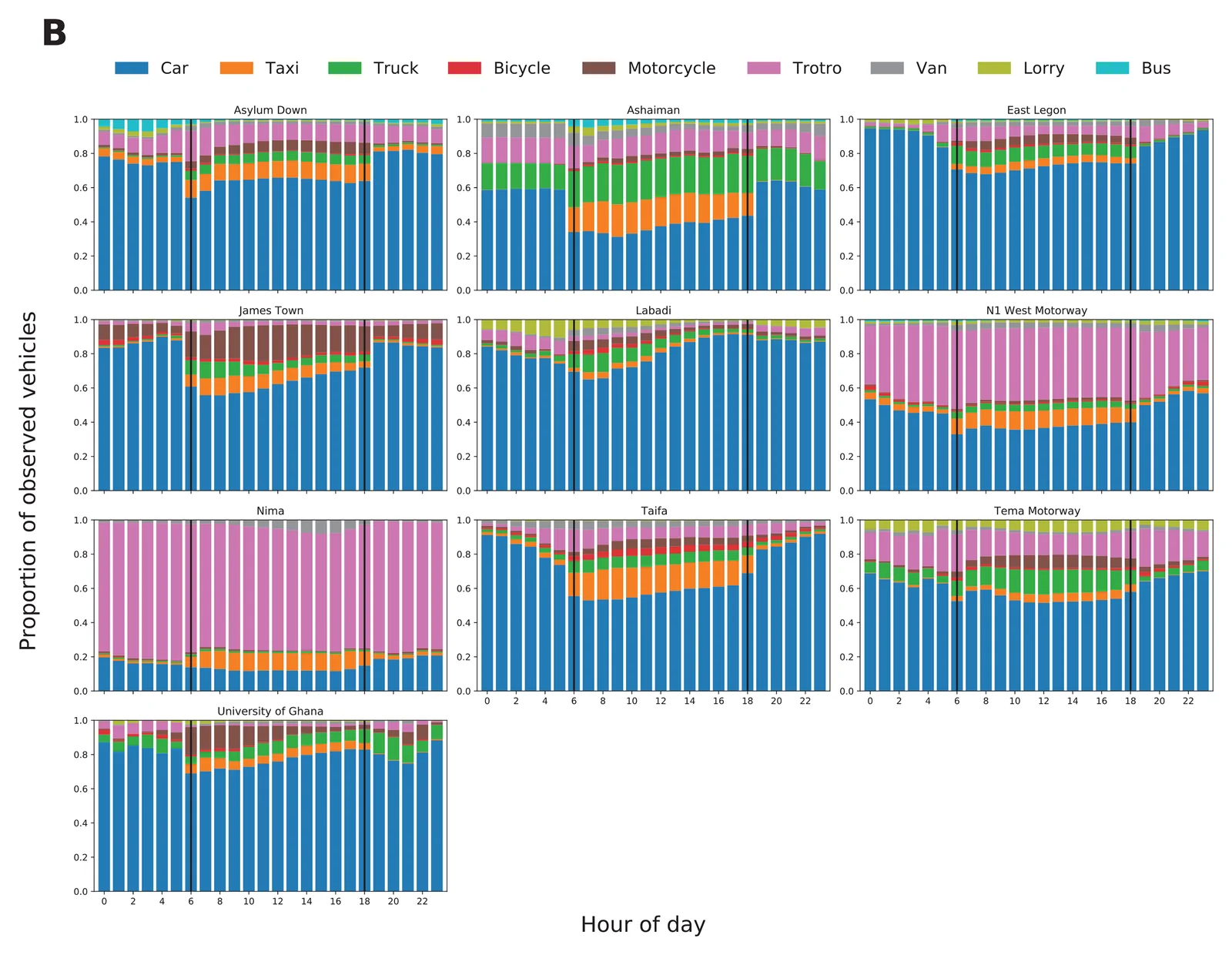


## Data Availability

Our analysis code, trained models, object count data and site metadata can be downloaded from http://globalenvhealth.org/code-data-download/ and http://equitablehealthycities.org/data-download/ upon publication of the paper. Requests for re-analysis of images should be sent to the corresponding author.
