# Supplementary material for: Capturing urban activities and environment across time scales: Object detection and time-series analysis of 6.8 million images in Accra, Ghana
Source: Cities. Author manuscript; Available in PMC 2026 Oct 30. (PMC7619661; doi:10.1016/j.cities.2026.107203)
Supplement: Supplementary file 1 [file EMS216147-supplement-Supplementery_Information.pdf]

Supplementary Information

Title

Capturing urban activities and environment across time scales: object detection and time-series analysis of 6.8 million images in Accra, Ghana

Content

SI S1: Transfer learning, data augmentation and hyperparameters for YOLOv7 3

SI S2: Selection of hyperparameters YOLOv7 and model evaluation 5

Supplementary Table 1: YOLOv7 models performance 6

Supplementary Table 2: Performance of final object detection model for each object class 7

Supplementary Table 3: Performance of final object detection model for each object class on high illumination daytime (colour) images 9

Supplementary Table 4: Performance of final object detection model for each object class on low-light nighttime (greyscale) images 10

Supplementary Table 5: Comparative analysis of object detection model performance across scale categories 11

Supplementary Figure 1. End-to-end flowchart of image processing and statistical analysis 13

Supplementary Figure 2. Extent of missingness of images 14

Supplementary Figure 3 a, b, c. Confusion matrices for object detection performance 15

Supplementary Figure 4 a, b, c. Precision-Recall (PR) curves for multi object detection 16

Supplementary Figure 5. Misdetection and omission samples in example images 17

Supplementary Figure 6: Comparison of statistical model performance with and without interaction term between hour and day 18

SI References 19

SI S1: Transfer learning, data augmentation and hyperparameters for YOLOv7

Transfer learning is a technique where a pre-trained deep model is reused as a starting point to train and adapt for a new task (Yosinski et al., 2014). In the context of YOLOv7 (Wang et al., 2022), the initial layers would have learnt general features like edges, textures and shapes from MS COCO dataset, which contains a wide variety of objects. Transfer learning improves performance and accelerates training when data are limited, by using a smaller learning rate to fine-tune existing features to a particular application (Zhang et al. 2024).

We employed data augmentation to increase the size and diversity of training data. Data augmentation helps the model to better generalise to diverse target images and prevent overfitting, making it more robust to variations in real-world settings. The data augmentation techniques were adopted from the original YOLOv7 paper that includes (a) image scaling: randomly resizing images for the model to generalise better to different object sizes; however the aspect ratio is preserved to prevent distortion; (b) image translation: shifting the image horizontally or vertically by a certain no. of pixels, to make the model invariant to the position of objects; (c) image flipping: flipping the images horizontally and vertically to create mirror images, enhancing model robustness to different object orientations; (d) Random cropping: randomly cutting and pasting parts of an image onto another image, which improves model robustness to occlusions; (e) mosaic: combining multiple images into a single frame, exposing the model to diverse scenes; (f) colour and exposure: randomly varying hue, saturation, and exposure of an image, and converting images to greyscale to simulate different lighting conditions and colour variations.

We adopted key hyperparameters, including the initial learning rate (0.01), momentum (0.937), and weight decay (0.0005), from the original YOLOv7 paper. The models were trained on multiple NVIDIA Quadro RTX 6000 GPUs. The YOLOv7 W6 model served as our baseline. Subsequently, we experimented with more advanced models such as E6, D6 and E6E, which

progressively increased complexity in terms of model depth, computational resources, training duration and trainable parameters. However, despite the increased complexity, these larger models did not yield significant performance improvement as seen in SI 3, leading us to select the E6 model as our final choice. As discussed in the main paper, two configurations of the E6 model performed well on different object categories. To leverage their strengths, we deployed both models, each extracting specific subsets of objects for which the model's performance was highest during testing.

SI S2: Selection of hyperparameters for YOLOv7 and model evaluation

As stated in the main paper, hyperparameters for the model were chosen from training on a set of 750 images and evaluated on a validation set of 250 images. The metric used to evaluate model performance for parameter tuning and final model evaluation on the test set was mean average precision (mAP), which measures whether the network accurately identifies both the presence of an object and localises its location and size, as represented by its boundaries (Padilla et al. 2020; Padilla et al. 2021). A true positive is defined when the predicted bounding box overlaps with the ground truth box above a range of intersection-over-union (the overlapping area between two bounding boxes divided by the area of their union) thresholds, from 0.50 to 0.95 in 0.05 intervals, and is identified as the correct object category. Identification is defined as when the final layer object classifier produces a confidence score above a specified threshold for a given object category. Precision is defined as the proportion of bounding boxes that are true positives, while recall is the fraction of true-positive detections as a proportion of all ground truth boxes. The average precision is the area under the precision-recall curve, obtained from varying the confidence score thresholds. The mean refers to the average taken across different intersection-over-union overlap thresholds, and confidence scores either for individual object categories or across object categories. This metric was originally constructed to evaluate the performance of diverse models on the MS-COCO dataset (Lin et al., 2015).

When applying the final model to our images, we employed non-maximum suppression (NMS), which removes the bounding box with the lower confidence score if two bounding boxes from the same class overlap more than 65% (the threshold adopted from YOLOv7). Additionally, we filter bounding boxes by selecting only those that exceed the confidence score threshold of 0.25 to qualify as final object detections. This process ensures that only the most confident prediction is retained for each object with dense predictions, effectively reducing redundancy.

Supplementary Table 1: YOLOv7 models training parameters

Model	Image size	Batch size	no. of model parameters	Minimum no. of GPUs x size required	Average time per epoch	Epoch no. for best result while training
W6 (baseline)	1280 x 1280	16	70.4 M	2 x 24 GB	45 sec	75
E6 (configuration-1)	1920 x 1920	16	97.2 M	8 x 24 GB	1 min	79
E6 (configuration-2)	1920 x 1920	8	97.2 M	4 x 24 GB	1 min	124

Supplementary Table 2: Performance of final object detection model for each object class

Object class	Counts in combined training and validation set (1000 images)	Counts in test set (250 images)	Chosen Model configuration	AP @.5:.95	AP @.5
Person	3331	855	E6-C2	0.464	0.749
Car	1302	336	E6-C1	0.455	0.658
Trash	826	211	E6-C1	0.116	0.213
Tro-tro	551	139	E6-C1	0.505	0.734
Debris	510	128	E6-C2	0.0454	0.0825
Umbrella	478	139	E6-C2	0.574	0.783
Taxi	363	85	E6-C2	0.612	0.746
Cooking bowl/pot	143	34	E6-C1	0.177	0.36
Pick-up truck	140	34	E6-C1	0.26	0.373
Market stall	133	35	E6-C2	0.105	0.198
Food	128	32	E6-C2	0.155	0.339
Motorcycle	126	31	E6-C2	0.382	0.645
Lorry	107	28	E6-C1	0.361	0.501
Van	105	26	E6-C2	0.0376	0.0541
Street vendor	97	22	E6-C2	0.073	0.129
Animal	74	19	E6-C2	0.326	0.48
Bicycle	53	13	E6-C2	0.322	0.57
Bus	20	5	E6-C2	0.385	0.584
Cookstove	14	4	E6-C1	0	0
Loudspeaker	13	4	E6-C1	0.434	0.62
**Total (weighted average)**	NA	NA	NA	**0.387**	**0.594**
**Total (simple average): mAP**	8,514	2,180	NA	**0.289**	**0.441**

‡ Average precision was calculated as described in SI S3; AP@.5:.95 is with varying intersection-over-union thresholds, starting from 0.5 to 0.95 with increments of 0.05, while AP@.5 is a single intersection-over-union with a threshold of 0.5

‡ Weighted average was used to mitigate the impact of imbalanced object class frequencies on the evaluation metric and to ensure the prevalence of each class is accounted appropriately. The weight was computed by dividing the number of objects for that particular class by the total number of all object classes across the entire dataset.

Supplementary Table 3: Performance of final object detection model for each object class on daytime (high-illumination) colour images

Object class	Counts in combined training and validation set (1000 images)	Counts in test set (126 images)	Chosen Model configuration	AP@.5:.95	AP@.5
Person	3331	656	E6- C2	0.469	0.749
Car	1302	234	E6- C1	0.501	0.716
Trash	826	163	E6- C1	0.088	0.169
Tro-tro	551	103	E6- C1	0.51	0.743
Debris	510	106	E6- C2	0.035	0.065
Umbrella	478	136	E6- C2	0.577	0.787
Taxi	363	66	E6- C2	0.691	0.835
Cooking bowl/pot	143	24	E6- C1	0.207	0.419
Pick-up truck	140	30	E6- C1	0.302	0.437
Market stall	133	26	E6- C2	0.156	0.295
Food	128	20	E6- C2	0.074	0.178
Motorcycle	126	27	E6- C2	0.393	0.658
Lorry	107	21	E6- C1	0.332	0.448
Van	105	22	E6- C2	0.009	0.02
Street vendor	97	17	E6- C2	0.107	0.193
Animal	74	14	E6- C2	0.327	0.451
Bicycle	53	12	E6- C2	0.34	0.62
Bus	20	4	E6- C2	0.253	0.459
Cookstove	14	3	E6- C1	0	0
Loudspeaker	13	3	E6- C1	0.581	0.83
**Total (weighted average)**	**NA**	**NA**	**NA**	**0.397**	**0.602**
**Total (simple average)**	**8,514**	**1,687**	**NA**	**0.298**	**0.454**

Supplementary Table 4: Performance of final object detection model for each object class on nighttime (low-light) greyscale images

Object class	Counts in combined training and validation set (1000 images)	Counts in test set (124 images)	Chosen Model configuration	AP@.5:.95	AP@.5
Person	3331	199	E6- C2	0.452	0.751
Car	1302	102	E6- C1	0.362	0.541
Trash	826	48	E6- C1	0.234	0.389
Tro-tro	551	36	E6- C1	0.503	0.728
Debris	510	22	E6- C2	0.111	0.187
Umbrella	478	3	E6- C2	0.533	0.665
Taxi	363	19	E6- C2	0.331	0.423
Cooking bowl/pot	143	10	E6- C1	0.119	0.248
Pick-up truck	140	4	E6- C1	0.007	0.009
Market stall	133	9	E6- C2	0.002	0.004
Food	128	12	E6- C2	0.277	0.574
Motorcycle	126	4	E6- C2	0.396	0.745
Lorry	107	7	E6- C1	0.544	0.785
Van	105	4	E6- C2	0.227	0.284
Street vendor	97	4	E6- C2	0	0
Animal	74	5	E6- C2	0.362	0.595
Bicycle	53	1	E6- C2	0	0
Bus	20	1	E6- C2	0.995	0.995
Cookstove	14	1	E6- C1	0	0
Loudspeaker	13	1	E6- C1	0	0
**Total (weighted average)**	**NA**	**NA**	**NA**	**0.366**	**0.583**
**Total (simple average)**	**8,514**	**492**	**NA**	**0.273**	**0.396**

Supplementary Table 5: Object detection model performance across object scale categories

Object class	Small object category		Medium object category		Large object category	
	Counts in testing set (250 images)	AP@.5	Counts in test set (250 images)	AP@.5	Counts in testing set (250 images)	AP@.5
Person	176	0.121	510	0.504	169	0.515
Car	19	0.028	155	0.296	162	0.496
Trash	163	0.147	47	0.233	1	0.012
Tro-tro	1	0.006	33	0.119	105	0.687
Debris	68	0.016	54	0.095	6	0.168
Umbrella	20	0.058	82	0.567	37	0.484
Taxi	0	0	26	0.203	59	0.658
Cooking bowl/pot	14	0.15	20	0.27	0	0
Pick-up truck	0	0	11	0.085	23	0.382
Market stall	0	0	16	0.115	19	0.152
Food	10	0.012	13	0.208	9	0.413
Motorcycle	3	0.043	18	0.467	10	0.331
Lorry	0	0	5	0.039	23	0.535
Van	0	0	11	0.004	15	0.083
Street vendor	2	0	14	0.07	5	0.177
Animal	10	0.31	8	0.248	1	0.249
Bicycle	3	0.085	8	0.566	2	0.248
Bus	0	0	1	0.166	4	0.578
Cookstove	1	0	3	0	0	0
Loudspeaker	2	0.495	2	0.414	0	0
**Total (weighted average)**	**492**	**0.111**	**1037**	**0.388**	**650**	**0.513**
**Total (simple average)**	**492**	**0.074**	**1037**	**0.234**	**650**	**0.308**

This table presents performance breakdown of consolidated object detection models across three object scale categories defined by MS-COCO dataset standards (Lin et al., 2015). The objects with an area less than 32^2 (1024 pixels) were categorised as small scale, area greater than 96^2 (9216 pixels) as large and rest as medium scale objects. The analysis compared the mean Average Precision at 0.5 IoU threshold (mAP@.5) across three object scale categories. The results indicate a relationship between object scale and detection accuracy, with performance with mean Average Precision at 0.5 IoU threshold (mAP@.5) increasing from 0.111 for small objects, 0.388 for medium objects to 0.513 for large objects.

Supplementary Figure 1. End-to-end flowchart of image processing and statistical analysis

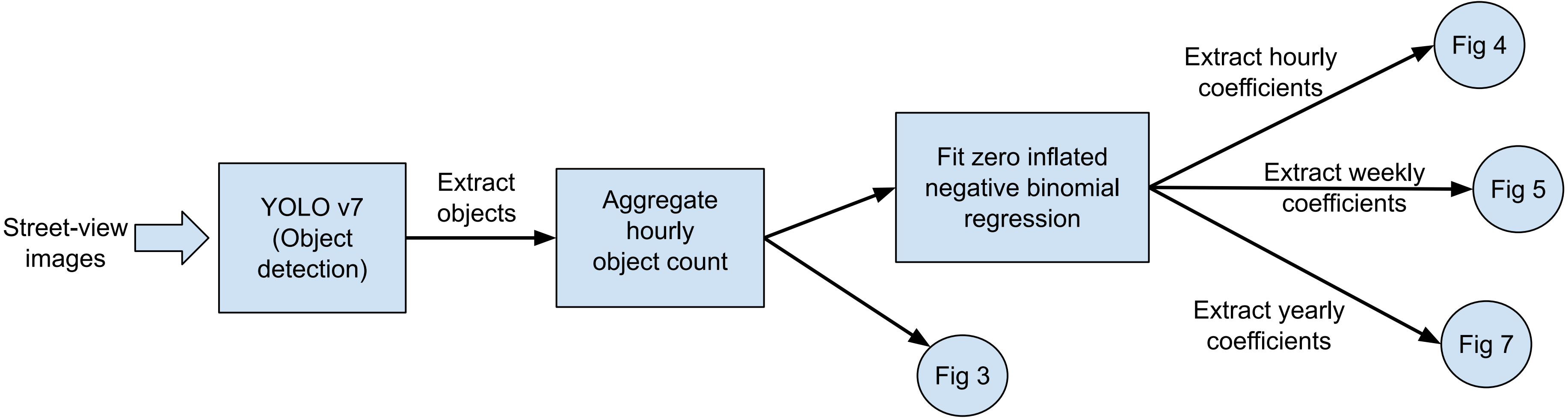
Street-view images
YOLO v7 (Object detection)
Extract objects
Aggregate hourly object count
Fig 3
Fit zero inflated negative binomial regression
Extract hourly coefficients
Fig 4
Extract weekly coefficients
Fig 5
Extract yearly coefficients
Fig 7

Supplementary Figure 2. Extent of missingness of images, by site

For each site, the figure shows the proportion of the target measurement period with zero, one or two cameras (for sites with two cameras) and zero or one camera (for sites with one camera).

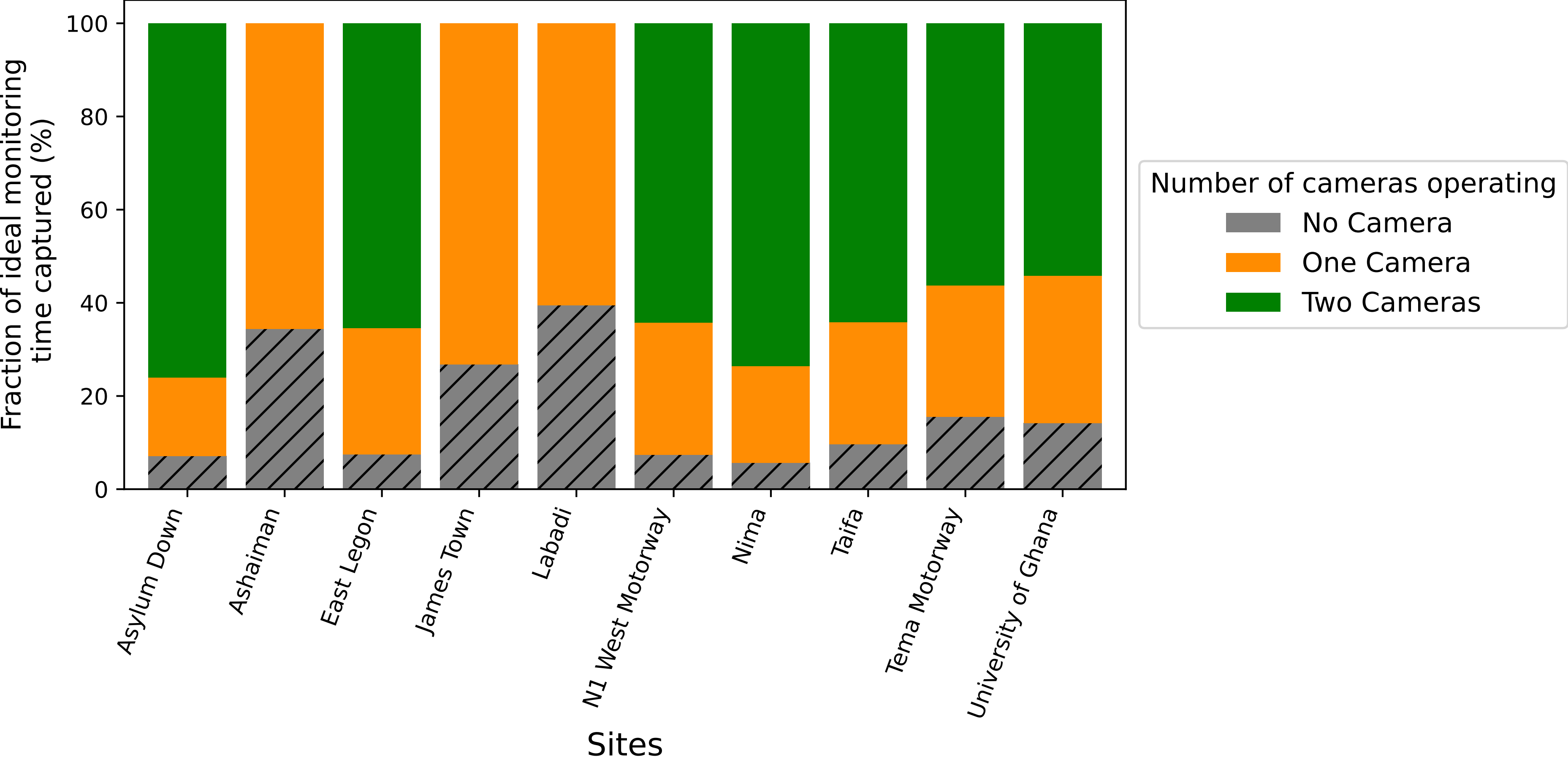

Fraction of ideal monitoring time captured (%)
0
20
40
60
80
100
Asylum Down
Ashaiman
East Legon
James Town
Labadi
N1 West Motorway
Nima
Taifa
Tema Motorway
University of Ghana
Sites
Number of cameras operating
No Camera
One Camera
Two Cameras

Supplementary Figure 3. Confusion matrices for object detection performance

Confusion matrix represents the classification accuracy and error types of consolidated object detection models. The rows represent the ground truth (actual) labels, while columns represent the predicted labels. The diagonal elements indicate the true positive and off-diagonal elements indicate misclassifications. Background False Positives represent wrongly detected cases and the background False Negatives represent the missed detected cases.

A) Aggregated Performance: Results from complete test dataset (250 images)

B) Daytime conditions: Results on daytime (high-illumination) colour (126 images)

C) Nighttime conditions: Results on nighttime (low-light) greyscale (124 images)

A

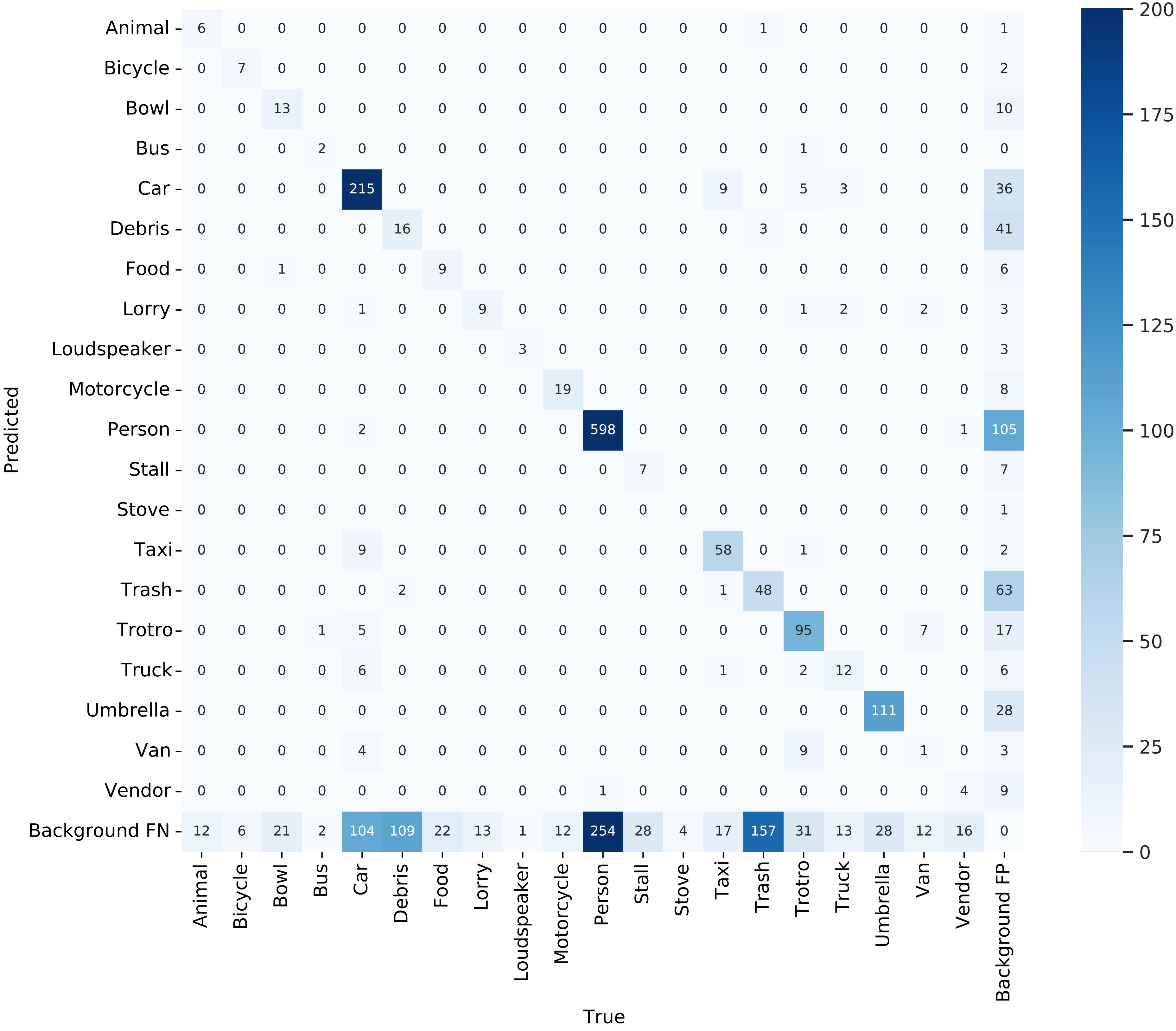

Animal 6 0 0 0 0 0 0 0 0 0 0 0 0 0 1 0 0 0 0 0 1
Bicycle 0 7 0 0 0 0 0 0 0 0 0 0 0 0 0 0 0 0 0 0 2
Bowl 0 0 13 0 0 0 0 0 0 0 0 0 0 0 0 0 0 0 0 0 10
Bus 0 0 0 2 0 0 0 0 0 0 0 0 0 0 0 1 0 0 0 0 0
Car 0 0 0 0 215 0 0 0 0 0 0 0 0 9 0 5 3 0 0 0 36
Debris 0 0 0 0 0 16 0 0 0 0 0 0 0 0 3 0 0 0 0 0 41
Food 0 0 1 0 0 0 9 0 0 0 0 0 0 0 0 0 0 0 0 0 6
Lorry 0 0 0 0 1 0 0 9 0 0 0 0 0 0 0 1 2 0 2 0 3
Loudspeaker 0 0 0 0 0 0 0 0 3 0 0 0 0 0 0 0 0 0 0 0 3
Motorcycle 0 0 0 0 0 0 0 0 0 19 0 0 0 0 0 0 0 0 0 0 8
Person 0 0 0 0 2 0 0 0 0 0 598 0 0 0 0 0 0 0 0 1 105
Stall 0 0 0 0 0 0 0 0 0 0 0 7 0 0 0 0 0 0 0 0 7
Stove 0 0 0 0 0 0 0 0 0 0 0 0 0 0 0 0 0 0 0 0 1
Taxi 0 0 0 0 9 0 0 0 0 0 0 0 0 58 0 1 0 0 0 0 2
Trash 0 0 0 0 0 2 0 0 0 0 0 0 0 1 48 0 0 0 0 0 63
Trotro 0 0 0 1 5 0 0 0 0 0 0 0 0 0 0 95 0 0 7 0 17
Truck 0 0 0 0 6 0 0 0 0 0 0 0 0 1 0 2 12 0 0 0 6
Umbrella 0 0 0 0 0 0 0 0 0 0 0 0 0 0 0 0 0 111 0 0 28
Van 0 0 0 0 4 0 0 0 0 0 0 0 0 0 0 9 0 0 1 0 3
Vendor 0 0 0 0 0 0 0 0 0 0 1 0 0 0 0 0 0 0 0 4 9
Background FN 12 6 21 2 104 109 22 13 1 12 254 28 4 17 157 31 13 28 12 16 0
Predicted
Animal Bicycle Bowl Bus Car Debris Food Lorry Loudspeaker Motorcycle Person Stall Stove Taxi Trash Trotro Truck Umbrella Van Vendor Background FP
True
200 175 150 125 100 75 50 25 0

B

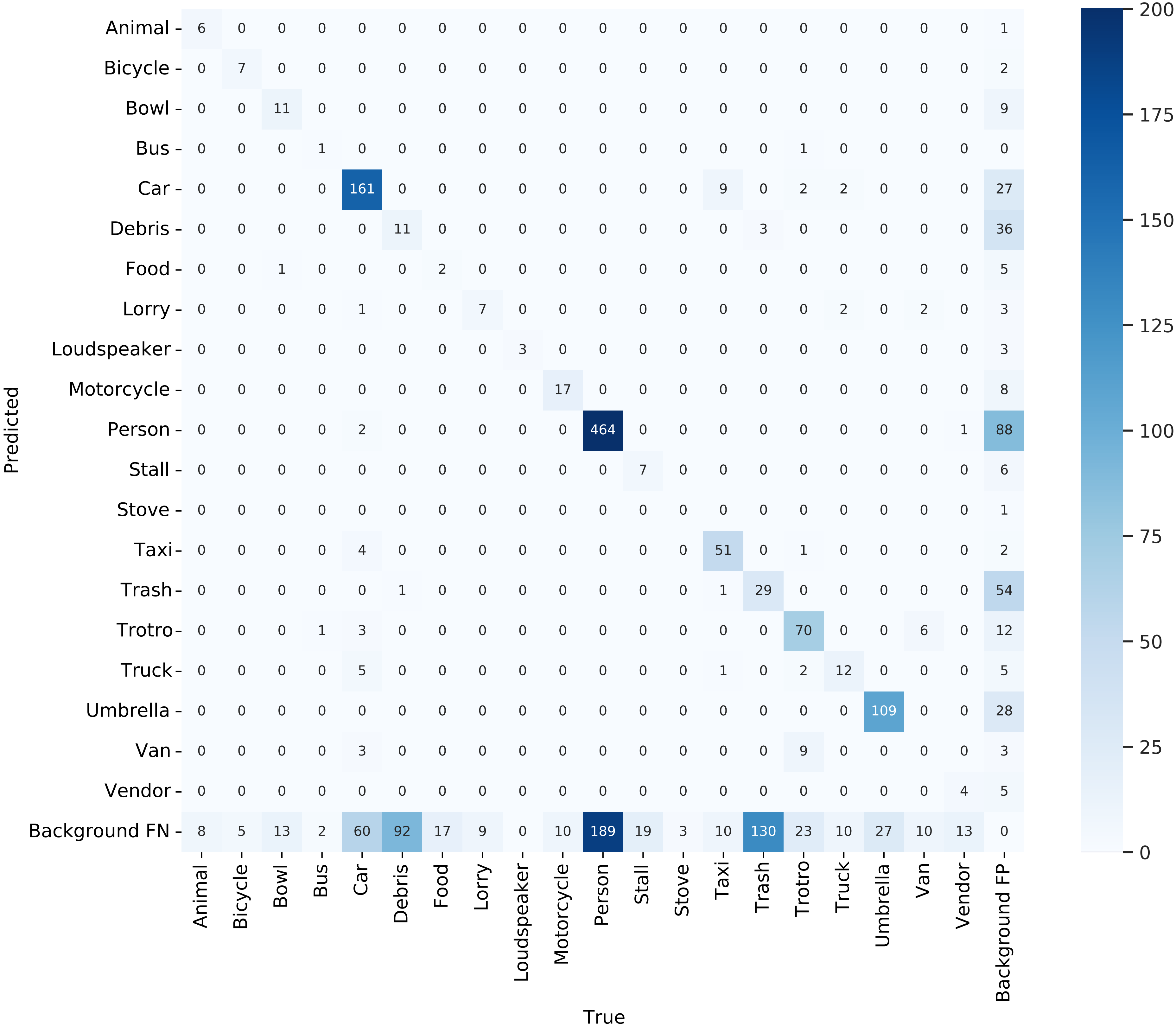
Animal 6 0 0 0 0 0 0 0 0 0 0 0 0 0 0 0 0 0 0 0 1
Bicycle 0 7 0 0 0 0 0 0 0 0 0 0 0 0 0 0 0 0 0 0 2
Bowl 0 0 11 0 0 0 0 0 0 0 0 0 0 0 0 0 0 0 0 0 9
Bus 0 0 0 1 0 0 0 0 0 0 0 0 0 0 0 1 0 0 0 0 0
Car 0 0 0 0 161 0 0 0 0 0 0 0 0 9 0 2 2 0 0 0 27
Debris 0 0 0 0 0 11 0 0 0 0 0 0 0 0 3 0 0 0 0 0 36
Food 0 0 1 0 0 0 2 0 0 0 0 0 0 0 0 0 0 0 0 0 5
Lorry 0 0 0 0 1 0 0 7 0 0 0 0 0 0 0 0 2 0 2 0 3
Loudspeaker 0 0 0 0 0 0 0 0 3 0 0 0 0 0 0 0 0 0 0 0 3
Motorcycle 0 0 0 0 0 0 0 0 0 17 0 0 0 0 0 0 0 0 0 0 8
Person 0 0 0 0 2 0 0 0 0 0 464 0 0 0 0 0 0 0 0 1 88
Stall 0 0 0 0 0 0 0 0 0 0 0 7 0 0 0 0 0 0 0 0 6
Stove 0 0 0 0 0 0 0 0 0 0 0 0 0 0 0 0 0 0 0 0 1
Taxi 0 0 0 0 4 0 0 0 0 0 0 0 0 51 0 1 0 0 0 0 2
Trash 0 0 0 0 0 1 0 0 0 0 0 0 0 1 29 0 0 0 0 0 54
Trotro 0 0 0 1 3 0 0 0 0 0 0 0 0 0 0 70 0 0 6 0 12
Truck 0 0 0 0 5 0 0 0 0 0 0 0 0 1 0 2 12 0 0 0 5
Umbrella 0 0 0 0 0 0 0 0 0 0 0 0 0 0 0 0 0 109 0 0 28
Van 0 0 0 0 3 0 0 0 0 0 0 0 0 0 0 9 0 0 0 0 3
Vendor 0 0 0 0 0 0 0 0 0 0 0 0 0 0 0 0 0 0 0 4 5
Background FN 8 5 13 2 60 92 17 9 0 10 189 19 3 10 130 23 10 27 10 13 0
Predicted
Animal Bicycle Bowl Bus Car Debris Food Lorry Loudspeaker Motorcycle Person Stall Stove Taxi Trash Trotro Truck Umbrella Van Vendor Background FP
True
200 175 150 125 100 75 50 25 0

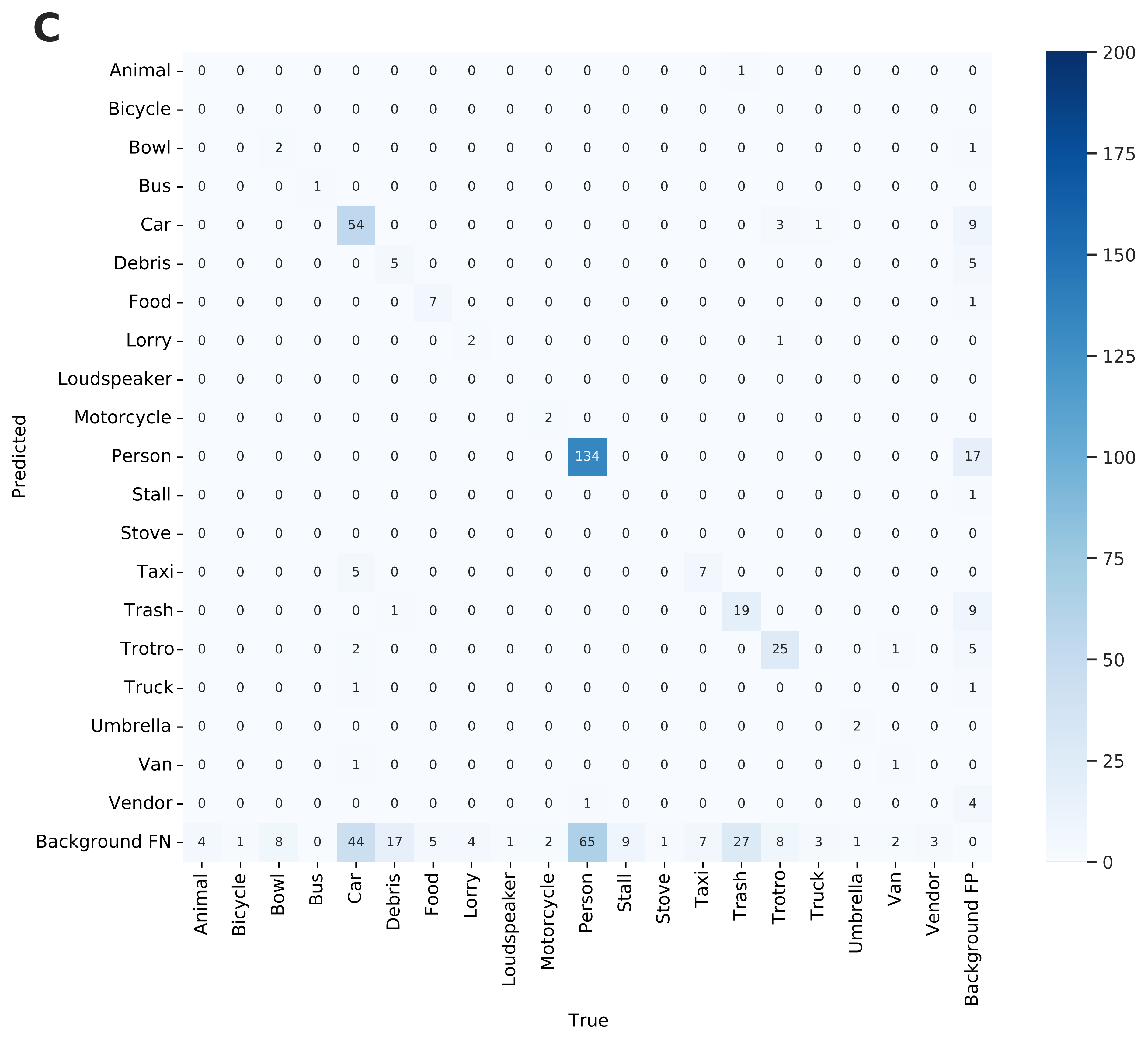
C
Predicted
True
Animal 0 0 0 0 0 0 0 0 0 0 0 0 0 0 1 0 0 0 0 0 0
Bicycle 0 0 0 0 0 0 0 0 0 0 0 0 0 0 0 0 0 0 0 0 0
Bowl 0 0 2 0 0 0 0 0 0 0 0 0 0 0 0 0 0 0 0 0 1
Bus 0 0 0 1 0 0 0 0 0 0 0 0 0 0 0 0 0 0 0 0 0
Car 0 0 0 0 54 0 0 0 0 0 0 0 0 0 0 3 1 0 0 0 9
Debris 0 0 0 0 0 5 0 0 0 0 0 0 0 0 0 0 0 0 0 0 5
Food 0 0 0 0 0 0 7 0 0 0 0 0 0 0 0 0 0 0 0 0 1
Lorry 0 0 0 0 0 0 0 2 0 0 0 0 0 0 0 1 0 0 0 0 0
Loudspeaker 0 0 0 0 0 0 0 0 0 0 0 0 0 0 0 0 0 0 0 0 0
Motorcycle 0 0 0 0 0 0 0 0 0 2 0 0 0 0 0 0 0 0 0 0 0
Person 0 0 0 0 0 0 0 0 0 0 134 0 0 0 0 0 0 0 0 0 17
Stall 0 0 0 0 0 0 0 0 0 0 0 0 0 0 0 0 0 0 0 0 1
Stove 0 0 0 0 0 0 0 0 0 0 0 0 0 0 0 0 0 0 0 0 0
Taxi 0 0 0 0 5 0 0 0 0 0 0 0 0 7 0 0 0 0 0 0 0
Trash 0 0 0 0 0 1 0 0 0 0 0 0 0 0 19 0 0 0 0 0 9
Trotro 0 0 0 0 2 0 0 0 0 0 0 0 0 0 0 25 0 0 1 0 5
Truck 0 0 0 0 1 0 0 0 0 0 0 0 0 0 0 0 0 0 0 0 1
Umbrella 0 0 0 0 0 0 0 0 0 0 0 0 0 0 0 0 0 2 0 0 0
Van 0 0 0 0 1 0 0 0 0 0 0 0 0 0 0 0 0 0 1 0 0
Vendor 0 0 0 0 0 0 0 0 0 0 1 0 0 0 0 0 0 0 0 0 4
Background FN 4 1 8 0 44 17 5 4 1 2 65 9 1 7 27 8 3 1 2 3 0
Animal Bicycle Bowl Bus Car Debris Food Lorry Loudspeaker Motorcycle Person Stall Stove Taxi Trash Trotro Truck Umbrella Van Vendor Background FP
200
175
150
125
100
75
50
25
0

Supplementary Figure 4. Precision-recall (PR) curves for multi object detection

PR curve represents the precision-recall trade off at a fixed IoU threshold of 0.5. The area under each curve represents the Average Precision (AP), a curve closer to the top right corner indicates superior balance of precision and recall. Each curve represents a specific class and the bold blue line represents Mean Average Precision (mAP@.5) across all categories.

A) Aggregated Performance: Results from complete test dataset (250 images)

B) Daytime conditions: Results on daytime (high-illumination) colour (126 images)

C) Nighttime conditions: Results on nighttime (low-light) greyscale (124 images)

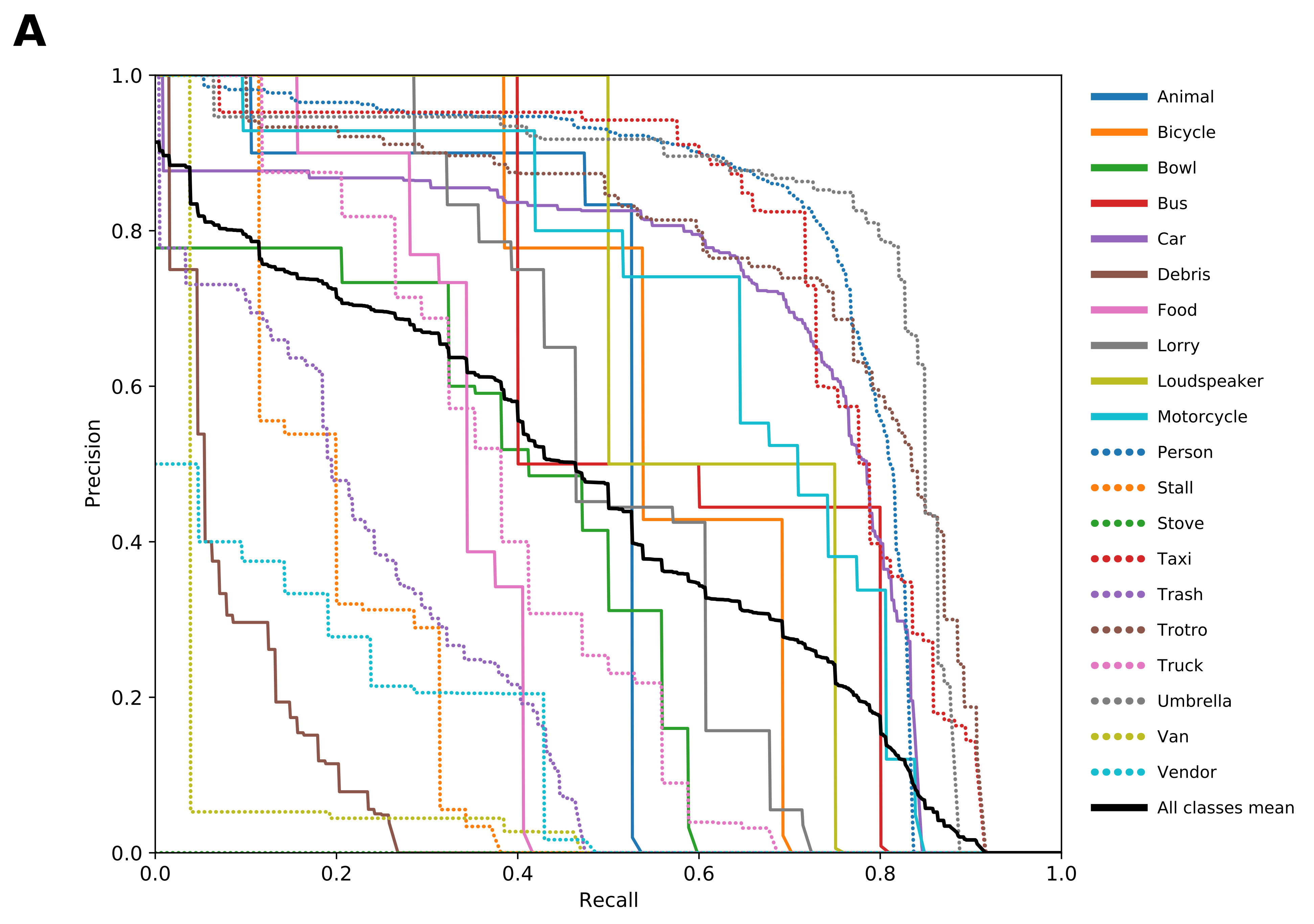
A
Precision
Recall
1.0
0.8
0.6
0.4
0.2
0.0
0.0
0.2
0.4
0.6
0.8
1.0
Animal
Bicycle
Bowl
Bus
Car
Debris
Food
Lorry
Loudspeaker
Motorcycle
Person
Stall
Stove
Taxi
Trash
Trotro
Truck
Umbrella
Van
Vendor
All classes mean

B

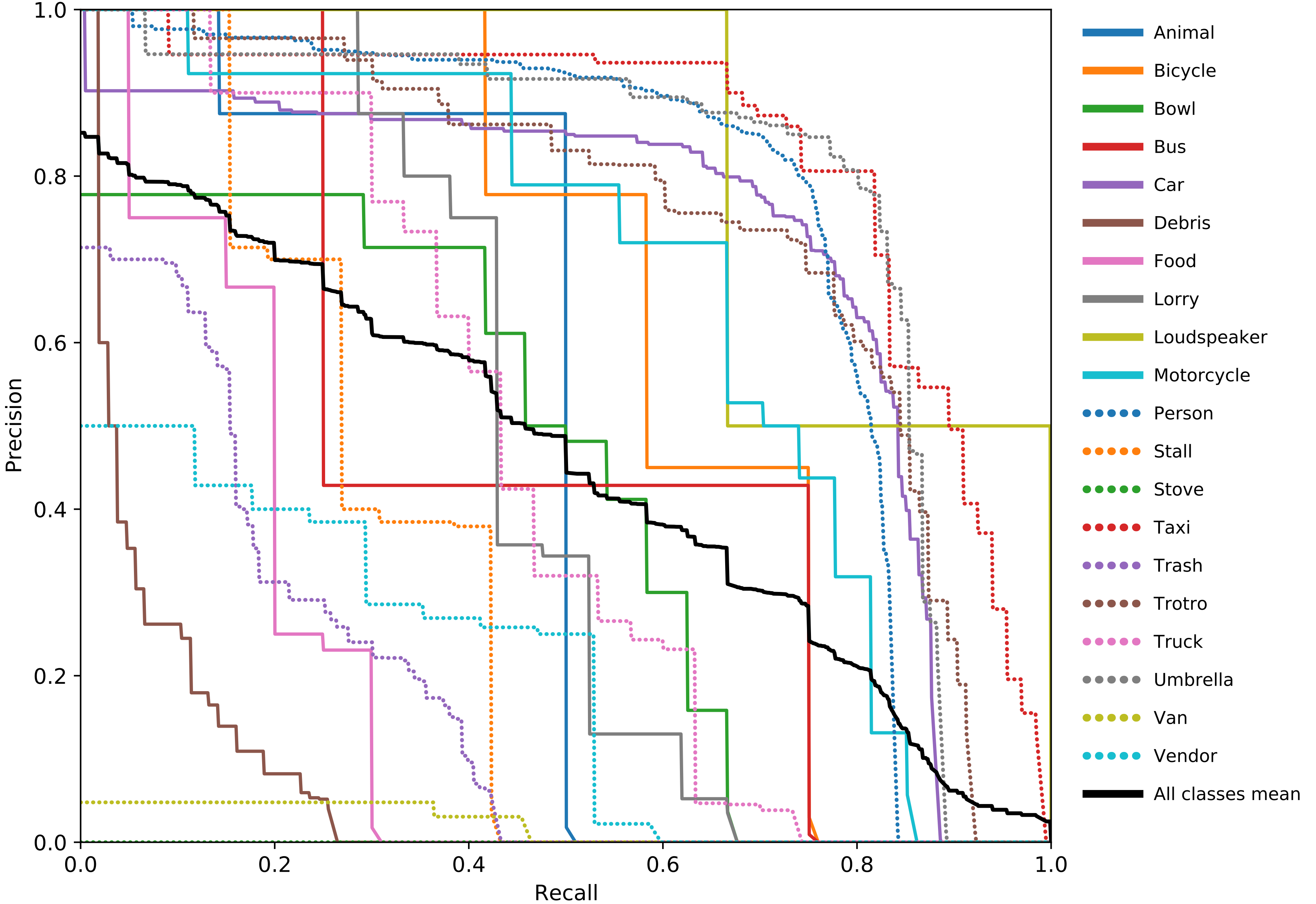

Precision
Recall
1.0
0.8
0.6
0.4
0.2
0.0
0.0
0.2
0.4
0.6
0.8
1.0
Animal
Bicycle
Bowl
Bus
Car
Debris
Food
Lorry
Loudspeaker
Motorcycle
Person
Stall
Stove
Taxi
Trash
Trotro
Truck
Umbrella
Van
Vendor
All classes mean

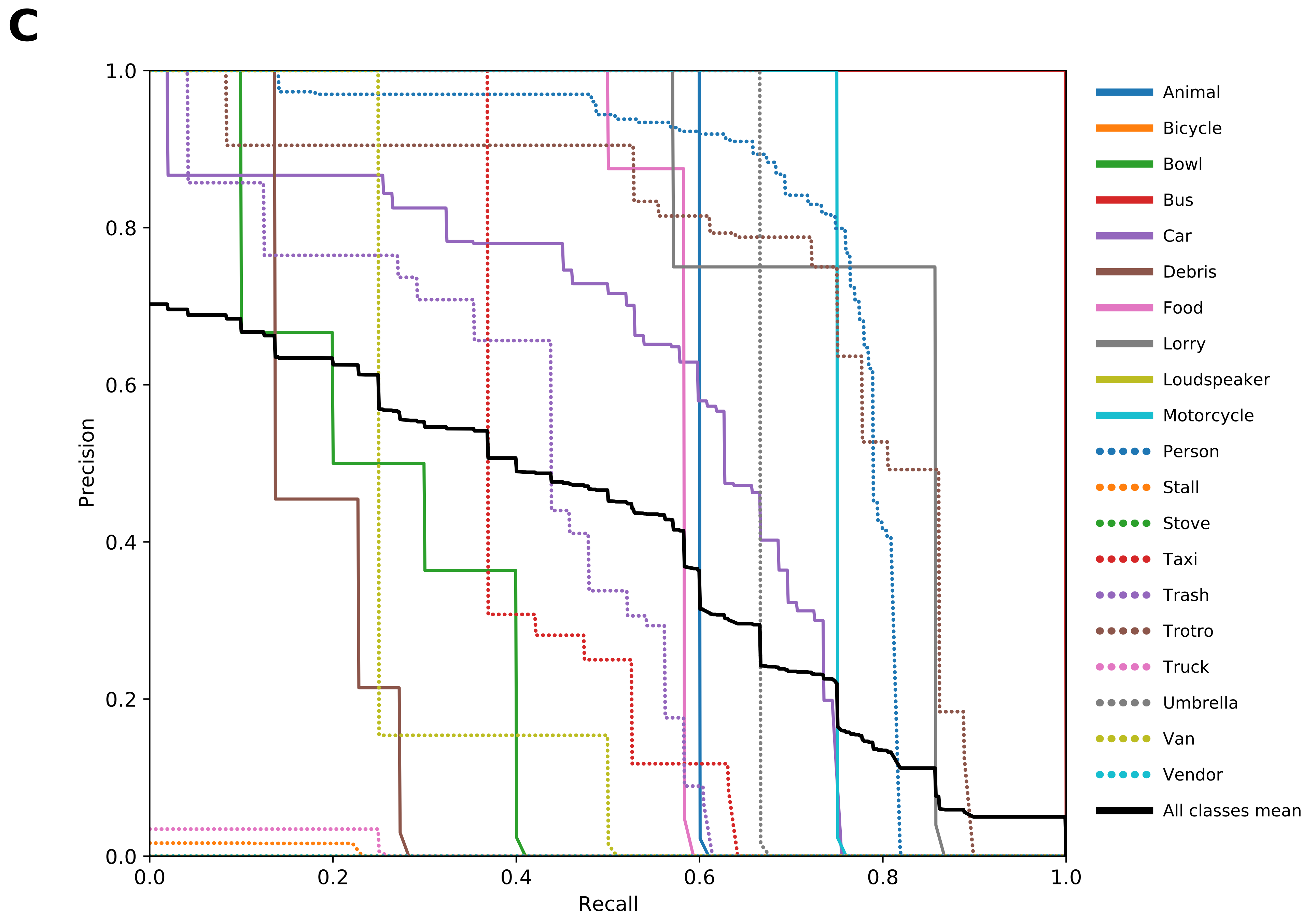
C
Precision
Recall
1.0
0.8
0.6
0.4
0.2
0.0
0.0
0.2
0.4
0.6
0.8
1.0
Animal
Bicycle
Bowl
Bus
Car
Debris
Food
Lorry
Loudspeaker
Motorcycle
Person
Stall
Stove
Taxi
Trash
Trotro
Truck
Umbrella
Van
Vendor
All classes mean

Supplementary Figure 5: Misdetection and omission samples in example images

This figure displays representative detection results across the sites. The left panel depicts the manual annotations (ground truth), while the right panel depicts the detection (model prediction). The samples highlight some model limitations. Instances where objects (e.g. people, market stalls, vehicles) are partially obscured by street activity. Challenges in detecting the small objects (e.g., debris, thrash) and limitations during low-light night (grey scale) images. Additionally, the model may miscategorise visually similar objects (e.g., car and taxi, person and vendor).

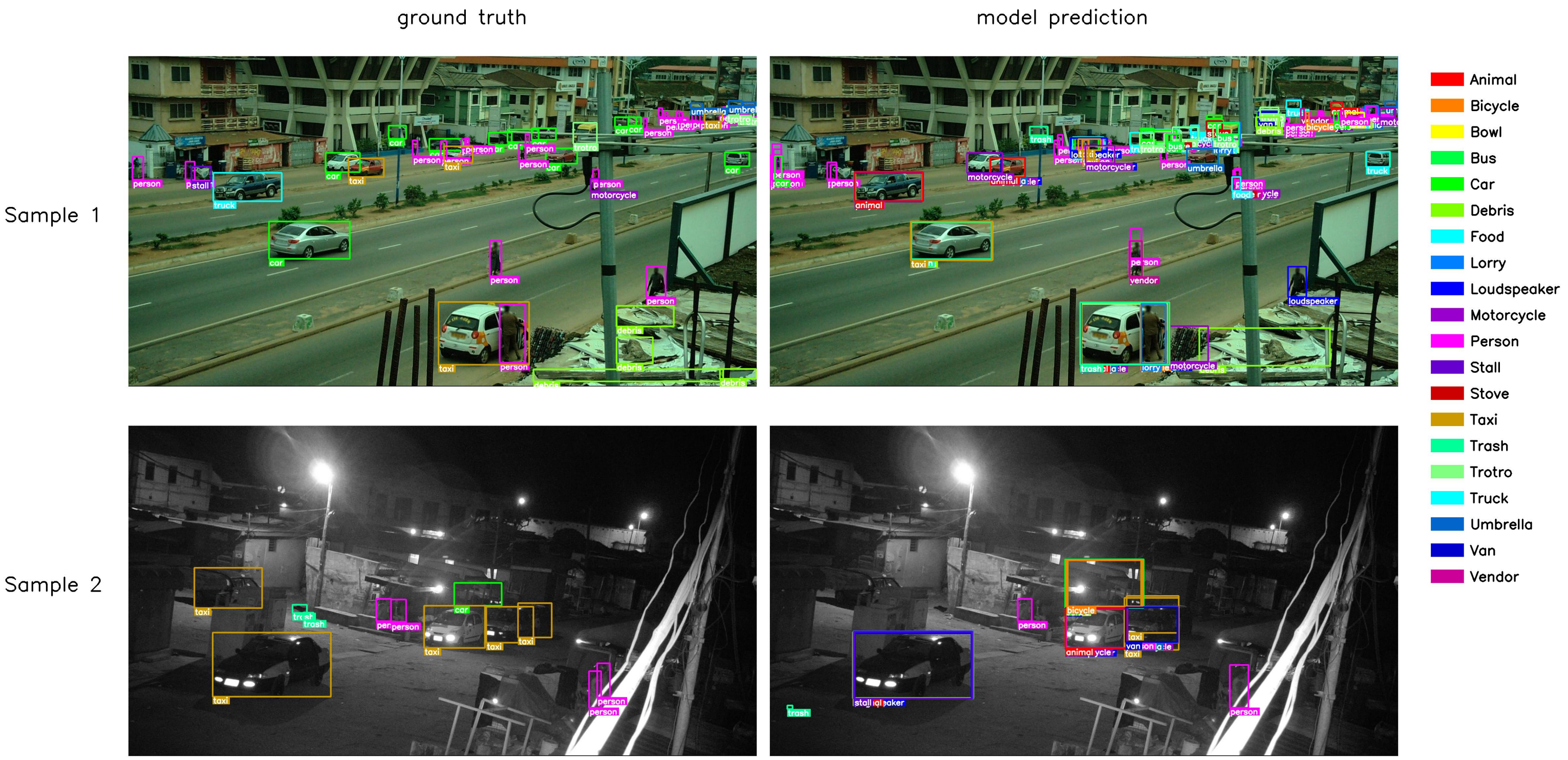
ground truth
model prediction
Sample 1
Sample 2
Animal
Bicycle
Bowl
Bus
Car
Debris
Food
Lorry
Loudspeaker
Motorcycle
Person
Stall
Stove
Taxi
Trash
Trotro
Truck
Umbrella
Van
Vendor

Supplementary Figure 6: Comparison of statistical model performance with and without interaction term between hour and day

The scatterplot compares the marginal (i.e., fixed-effect only) R^2 of models with and without interaction terms between hour-of-day and day-of-week variables in the model. X and Y axis values show the average R^2 value from five-fold cross-validation for the two models. Points above the diagonal line indicate greater variations in hourly patterns across different days of the week. As most points fall above or close to the diagonal line, this suggests that incorporating an interaction term between hour and day into the model improved its performance.

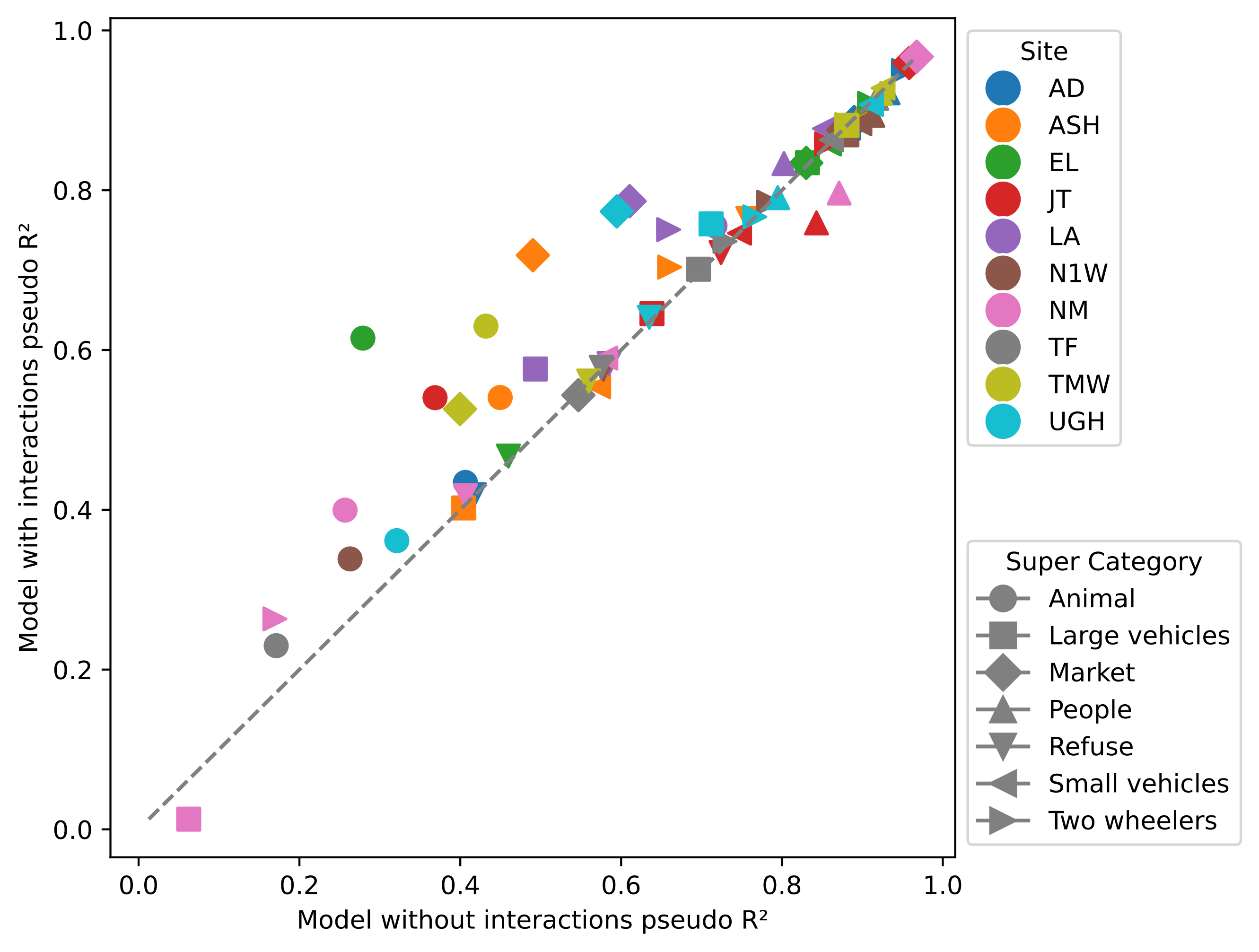

Model with interactions pseudo R²
Model without interactions pseudo R²
0.0
0.2
0.4
0.6
0.8
1.0
Site
AD
ASH
EL
JT
LA
N1W
NM
TF
TMW
UGH
Super Category
Animal
Large vehicles
Market
People
Refuse
Small vehicles
Two wheelers

SI References

Lin, T.-Y., Maire, M., Belongie, S., Bourdev, L., Girshick, R., Hays, J., Perona, P., Ramanan, D., Zitnick, C. L., & Dollár, P. (2015). *Microsoft COCO: Common Objects in Context* (No. arXiv:1405.0312). arXiv. https://doi.org/10.48550/arXiv.1405.0312

Padilla, R., Netto, S. L., & da Silva, E. A. B. (2020). A Survey on Performance Metrics for Object-Detection Algorithms. *2020 International Conference on Systems, Signals and Image Processing (IWSSIP)*, 237–242. https://doi.org/10.1109/IWSSIP48289.2020.9145130

Padilla, R., Passos, W. L., Dias, T. L. B., Netto, S. L., & da Silva, E. A. B. (2021). A Comparative Analysis of Object Detection Metrics with a Companion Open-Source Toolkit. *Electronics*, *10*(3), Article 3. https://doi.org/10.3390/electronics10030279

Wang, C.-Y., Bochkovskiy, A., & Liao, H.-Y. M. (2022). *YOLOv7: Trainable bag-of-freebies sets new state-of-the-art for real-time object detectors* (No. arXiv:2207.02696). arXiv. https://doi.org/10.48550/arXiv.2207.02696

Yosinski, J., Clune, J., Bengio, Y., & Lipson, H. (2014). *How transferable are features in deep neural networks?* https://doi.org/10.48550/ARXIV.1411.1792

Zhang, S. F., Hsu, S. J., Chen, C. H., & Cheng, R.-S. (2024). Object Detection Transfer Learning paradigm on Cross Domain Street Vehicle Detection. *Proceedings of the 2024 International Conference on Information Technology, Data Science, and Optimization*, 20–24. https://doi.org/10.1145/3658549.3658554
